# Rare deleterious mutations of *HNRNP* genes result in shared neurodevelopmental disorders

**DOI:** 10.1186/s13073-021-00870-6

**Published:** 2021-04-19

**Authors:** Madelyn A. Gillentine, Tianyun Wang, Kendra Hoekzema, Jill Rosenfeld, Pengfei Liu, Hui Guo, Chang N. Kim, Bert B. A. De Vries, Lisenka E. L. M. Vissers, Magnus Nordenskjold, Malin Kvarnung, Anna Lindstrand, Ann Nordgren, Jozef Gecz, Maria Iascone, Anna Cereda, Agnese Scatigno, Silvia Maitz, Ginevra Zanni, Enrico Bertini, Christiane Zweier, Sarah Schuhmann, Antje Wiesener, Micah Pepper, Heena Panjwani, Erin Torti, Farida Abid, Irina Anselm, Siddharth Srivastava, Paldeep Atwal, Carlos A. Bacino, Gifty Bhat, Katherine Cobian, Lynne M. Bird, Jennifer Friedman, Meredith S. Wright, Bert Callewaert, Florence Petit, Sophie Mathieu, Alexandra Afenjar, Celenie K. Christensen, Kerry M. White, Orly Elpeleg, Itai Berger, Edward J. Espineli, Christina Fagerberg, Charlotte Brasch-Andersen, Lars Kjærsgaard Hansen, Timothy Feyma, Susan Hughes, Isabelle Thiffault, Bonnie Sullivan, Shuang Yan, Kory Keller, Boris Keren, Cyril Mignot, Frank Kooy, Marije Meuwissen, Alice Basinger, Mary Kukolich, Meredith Philips, Lucia Ortega, Margaret Drummond-Borg, Mathilde Lauridsen, Kristina Sorensen, Anna Lehman, CAUSES Study, Elena Lopez-Rangel, Paul Levy, Davor Lessel, Timothy Lotze, Suneeta Madan-Khetarpal, Jessica Sebastian, Jodie Vento, Divya Vats, L. Manace Benman, Shane Mckee, Ghayda M. Mirzaa, Candace Muss, John Pappas, Hilde Peeters, Corrado Romano, Maurizio Elia, Ornella Galesi, Marleen E. H. Simon, Koen L. I. van Gassen, Kara Simpson, Robert Stratton, Sabeen Syed, Julien Thevenon, Irene Valenzuela Palafoll, Antonio Vitobello, Marie Bournez, Laurence Faivre, Kun Xia, SPARK Consortium, John Acampado, Andrea J. Ace, Alpha Amatya, Irina Astrovskaya, Asif Bashar, Elizabeth Brooks, Martin E. Butler, Lindsey A. Cartner, Wubin Chin, Wendy K. Chung, Amy M. Daniels, Pamela Feliciano, Chris Fleisch, Swami Ganesan, William Jensen, Alex E. Lash, Richard Marini, Vincent J. Myers, Eirene O’Connor, Chris Rigby, Beverly E. Robertson, Neelay Shah, Swapnil Shah, Emily Singer, Lee Anne G. Snyder, Alexandra N. Stephens, Jennifer Tjernagel, Brianna M. Vernoia, Natalia Volfovsky, Loran Casey White, Alexander Hsieh, Yufeng Shen, Xueya Zhou, Tychele N. Turner, Ethan Bahl, Taylor R. Thomas, Leo Brueggeman, Tanner Koomar, Jacob J. Michaelson, Brian J. O’Roak, Rebecca A. Barnard, Richard A. Gibbs, Donna Muzny, Aniko Sabo, Kelli L. Baalman Ahmed, Evan E. Eichler, Matthew Siegel, Leonard Abbeduto, David G. Amaral, Brittani A. Hilscher, Deana Li, Kaitlin Smith, Samantha Thompson, Charles Albright, Eric M. Butter, Sara Eldred, Nathan Hanna, Mark Jones, Daniel Lee Coury, Jessica Scherr, Taylor Pifher, Erin Roby, Brandy Dennis, Lorrin Higgins, Melissa Brown, Michael Alessandri, Anibal Gutierrez, Melissa N. Hale, Lynette M. Herbert, Hoa Lam Schneider, Giancarla David, Robert D. Annett, Dustin E. Sarver, Ivette Arriaga, Alexies Camba, Amanda C. Gulsrud, Monica Haley, James T. McCracken, Sophia Sandhu, Maira Tafolla, Wha S. Yang, Laura A. Carpenter, Catherine C. Bradley, Frampton Gwynette, Patricia Manning, Rebecca Shaffer, Carrie Thomas, Raphael A. Bernier, Emily A. Fox, Jennifer A. Gerdts, Micah Pepper, Theodore Ho, Daniel Cho, Joseph Piven, Holly Lechniak, Latha V. Soorya, Rachel Gordon, Allison Wainer, Lisa Yeh, Cesar Ochoa-Lubinoff, Nicole Russo, Elizabeth Berry-Kravis, Stephanie Booker, Craig A. Erickson, Lisa M. Prock, Katherine G. Pawlowski, Emily T. Matthews, Stephanie J. Brewster, Margaret A. Hojlo, Evi Abada, Elena Lamarche, Tianyun Wang, Shwetha C. Murali, William T. Harvey, Hannah E. Kaplan, Karen L. Pierce, Lindsey DeMarco, Susannah Horner, Juhi Pandey, Samantha Plate, Mustafa Sahin, Katherine D. Riley, Erin Carmody, Julia Constantini, Amy Esler, Ali Fatemi, Hanna Hutter, Rebecca J. Landa, Alexander P. McKenzie, Jason Neely, Vini Singh, Bonnie Van Metre, Ericka L. Wodka, Eric J. Fombonne, Lark Y. Huang-Storms, Lillian D. Pacheco, Sarah A. Mastel, Leigh A. Coppola, Sunday Francis, Andrea Jarrett, Suma Jacob, Natasha Lillie, Jaclyn Gunderson, Dalia Istephanous, Laura Simon, Ori Wasserberg, Angela L. Rachubinski, Cordelia R. Rosenberg, Stephen M. Kanne, Amanda D. Shocklee, Nicole Takahashi, Shelby L. Bridwell, Rebecca L. Klimczac, Melissa A. Mahurin, Hannah E. Cotrell, Cortaiga A. Grant, Samantha G. Hunter, Christa Lese Martin, Cora M. Taylor, Lauren K. Walsh, Katherine A. Dent, Andrew Mason, Anthony Sziklay, Christopher J. Smith, Rachel K. Earl, Tomasz Nowakowski, Raphael A. Bernier, Evan E. Eichler

**Affiliations:** 1 Department of Genome Sciences, University of Washington School of Medicine, 3720 15th Ave NE S413A, Box 355065, Seattle, WA 981095-5065 USA; 2 Baylor Genetics Laboratories, Houston, TX USA; 3 Department of Molecular & Human Genetics, Baylor College of Medicine, Houston, TX USA; 4 Center for Medical Genetics and Hunan Key Laboratory of Medical Genetics, School of Life Sciences, Central South University, Changsha, Hunan China; 5 Department of Anatomy, University of California, San Francisco, CA USA; 6 Department of Psychiatry, University of California, San Francisco, CA USA; 7 Weill Institute for Neurosciences, University of California at San Francisco, San Francisco, CA USA; 8 The Eli and Edythe Broad Center of Regeneration Medicine and Stem Cell Research, University of California, San Francisco, CA USA; 9 Department of Human Genetics, Radboud University Medical Center, Nijmegen, The Netherlands; 10 Department of Molecular Medicine and Surgery, Center for Molecular Medicine, Karolinska Institutet, Stockholm, Sweden; 11 Department of Clinical Genetics, Karolinska University Hospital, Stockholm, Sweden; 12 School of Medicine and the Robinson Research Institute, the University of Adelaide at the Women’s and Children’s Hospital, Adelaide, South Australia Australia; 13 Genetics and Molecular Pathology, SA Pathology, Adelaide, South Australia Australia; 14 South Australian Health and Medical Research Institute, Adelaide, South Australia Australia; 15 Laboratorio di Genetica Medica - ASST Papa Giovanni XXIII, Bergamo, Italy; 16 Department of Pediatrics, ASST Papa Giovanni XXIII, Bergamo, Italy; 17 Genetic Unit, Department of Pediatrics, Fondazione MBBM S. Gerardo Hospital, Monza, Italy; 18 Unit of Neuromuscular and Neurodegenerative Disorders, Department Neurosciences, Bambino Gesù Children’s Hospital, IRCCS, 00146 Rome, Italy; 19 Institute of Human Genetics, Friedrich-Alexander-Universität Erlangen-Nürnberg (FAU), Erlangen, Germany; 20 Center on Human Development and Disability, University of Washington, Seattle, WA USA; 21 Seattle Children’s Autism Center, Seattle, WA USA; 22 GeneDX, Gaithersburg, MD USA; 23 Department of Pediatrics-Neurology, Baylor College of Medicine, Houston, TX USA; 24 Texas Children’s Hospital, Houston, TX USA; 25 Department of Neurology, Boston Children’s Hospital, Harvard Medical School, Boston, MA USA; 26 The Atwal Clinic: Genomic & Personalized Medicine, Jacksonville, FL USA; 27 Department of Pediatrics, Section of Genetics, University of Illinois at Chicago, Chicago, IL USA; 28 Department of Pediatrics, University of California San Diego, San Diego, CA USA; 29 Genetics/Dysmorphology, Rady Children’s Hospital San Diego, San Diego, CA USA; 30 Rady Children’s Institute for Genomic Medicine, San Diego, CA USA; 31 Department of Neurosciences, University of California San Diego, San Diego, CA USA; 32 Department of Biomolecular Medicine, Ghent University Hospital, Ghent, Belgium; 33 Clinique de Génétique, Hôpital Jeanne de Flandre, Bâtiment Modulaire, CHU, 59037 Lille Cedex, France; 34 Sorbonne Universités, Centre de Référence déficiences intellectuelles de causes rares, département de génétique et embryologie médicale, Hôpital Trousseau, AP-HP, Paris, France; 35 Department of Pediatrics, Indiana University School of Medicine, Indianapolis, IN USA; 36 Department of Medical and Molecular Genetics, IU Health, Indianapolis, IN USA; 37 Department of Genetics, Hadassah, Hebrew University Medical Center, Jerusalem, Israel; 38 Pediatric Neurology, Assuta-Ashdod University Hospital, Ashdod, Israel; 39 Health Sciences, Ben-Gurion University of the Negev, Beersheba, Israel; 40 Department of Clinical Genetics, Odense University Hospital, Odense, Denmark; 41 H C Andersen Chilldrens Hospital, Odense University Hospital, Odense, Denmark; 42 Gillette Children’s Specialty Healthcare, Saint Paul, MN USA; 43 Division of Clinical Genetics, Children’s Mercy Kansas City, Kansas City, MO USA; 44 The University of Missouri-Kansas City, School of Medicine, Kansas City, MO USA; 45 Children’s Mercy Kansas City, Center for Pediatric Genomic Medicine, Kansas City, MO USA; 46 Oregon Health & Science University, Corvallis, OR USA; 47 Department of Genetics, Hópital Pitié-Salpêtrière, Paris, France; 48 Department of Medical Genetics, University of Antwerp, Antwerp, Belgium; 49 Genetics Department, Cook Children’s Hospital, Fort Worth, TX USA; 50 Department of Medical Genetics, University of British Columbia, Vancouver, Canada; 51 BC Children’s Hospital and BC Women’s Hospital, Vancouver, BC Canada; 52 Division of Developmental Pediatrics, Department of Pediatrics, BC Children’s Hospital, University of British Columbia, Vancouver, BC Canada; 53 Sunny Hill Health Centre for Children, Vancouver, BC Canada; 54 Department of Pediatrics, The Children’s Hospital at Montefiore, Bronx, NY USA; 55 Institute of Human Genetics, University Medical Center Hamburg-Eppendorf, Hamburg, Germany; 56 Department of Human Genetics, University of Pittsburgh, Pittsburgh, PA USA; 57 UPMC Children’s Hospital of Pittsburgh, Pittsburgh, PA USA; 58 Kaiser Permanente Southern California, Los Angeles, CA USA; 59 The Permanente Medical Group, Oakland, CA USA; 60 Northern Ireland Regional Genetics Service, Belfast City Hospital, Belfast, UK; 61 Center for Integrative Brain Research, Seattle Children’s Research Institute, Seattle, WA USA; 62 Department of Pediatrics, University of Washington, Seattle, WA USA; 63 Brotman Baty Institute for Precision Medicine, Seattle, WA USA; 64 Al Dupont Hospital for Children, Wilmington, DE USA; 65 NYU Grossman School of Medicine, Department of Pediatrics, Clinical Genetic Services, New York, NY USA; 66 Center for Human Genetics, KU Leuven and Leuven Autism Research (LAuRes), Leuven, Belgium; 67 Oasi Research Institute-IRCCS, Troina, Italy; 68 Department of Genetics, University Medical Center, Utrecht University, Utrecht, The Netherlands; 69 Rare Disease Institute, Children’s National Health System, Washington, DC USA; 70 Department of Genetics, Driscoll Children’s Hospital, Corpus Christi, TX USA; 71 Department of Pediatric Gastroenterology, Driscoll Children’s Hospital, Corpus Christi, TX USA; 72 Àrea de Genètica Clínica i Molecular, Hospital Vall d’Hebrón, Barcelona, Spain; 73 Centre de référence Anomalies du développement, CHU Grenoble-Alpes, Grenoble, France; 74 UF Innovation en Diagnostic Génomique des Maladies Rares, FHU-TRANSLAD, CHU Dijon Bourgogne and INSERM UMR1231 GAD, Université de Bourgogne Franche-Comté, F-21000 Dijon, France; 75 INSERM UMR 1231 Génétique des Anomalies du Développement, Université Bourgogne Franche-Comté, Dijon, France; 76 Centre de Référence Maladies Rares « déficience intellectuelle », Centre de Génétique, FHU-TRANSLAD, CHU Dijon Bourgogne, Dijon, France; 77 Centre de Référence Maladies Rares « Anomalies du Développement et Syndromes malformatifs »​ Université Bourgogne Franche-Comté, Dijon, France; 78 Department of Psychiatry and Behavioral Sciences, University of Washington, Seattle, WA USA; 79 Howard Hughes Medical Institute, University of Washington, Seattle, WA USA

**Keywords:** Neurodevelopmental disorders, hnRNPs, Cortex development, Gene families

## Abstract

**Background:**

With the increasing number of genomic sequencing studies, hundreds of genes have been implicated in neurodevelopmental disorders (NDDs). The rate of gene discovery far outpaces our understanding of genotype–phenotype correlations, with clinical characterization remaining a bottleneck for understanding NDDs. Most disease-associated Mendelian genes are members of gene families, and we hypothesize that those with related molecular function share clinical presentations.

**Methods:**

We tested our hypothesis by considering gene families that have multiple members with an enrichment of de novo variants among NDDs, as determined by previous meta-analyses. One of these gene families is the heterogeneous nuclear ribonucleoproteins (hnRNPs), which has 33 members, five of which have been recently identified as NDD genes (*HNRNPK*, *HNRNPU*, *HNRNPH1*, *HNRNPH2*, and *HNRNPR*) and two of which have significant enrichment in our previous meta-analysis of probands with NDDs (*HNRNPU* and *SYNCRIP*). Utilizing protein homology, mutation analyses, gene expression analyses, and phenotypic characterization, we provide evidence for variation in 12 *HNRNP* genes as candidates for NDDs. Seven are potentially novel while the remaining genes in the family likely do not significantly contribute to NDD risk.

**Results:**

We report 119 new NDD cases (64 de novo variants) through sequencing and international collaborations and combined with published clinical case reports. We consider 235 cases with gene-disruptive single-nucleotide variants or indels and 15 cases with small copy number variants. Three hnRNP-encoding genes reach nominal or exome-wide significance for de novo variant enrichment, while nine are candidates for pathogenic mutations. Comparison of *HNRNP* gene expression shows a pattern consistent with a role in cerebral cortical development with enriched expression among radial glial progenitors. Clinical assessment of probands (*n* = 188–221) expands the phenotypes associated with *HNRNP* rare variants, and phenotypes associated with variation in the *HNRNP* genes distinguishes them as a subgroup of NDDs.

**Conclusions:**

Overall, our novel approach of exploiting gene families in NDDs identifies new *HNRNP*-related disorders, expands the phenotypes of known *HNRNP*-related disorders, strongly implicates disruption of the hnRNPs as a whole in NDDs, and supports that NDD subtypes likely have shared molecular pathogenesis. To date, this is the first study to identify novel genetic disorders based on the presence of disorders in related genes. We also perform the first phenotypic analyses focusing on related genes. Finally, we show that radial glial expression of these genes is likely critical during neurodevelopment. This is important for diagnostics, as well as developing strategies to best study these genes for the development of therapeutics.

## Background

Among the hundreds of candidate genes proposed for neurodevelopmental disorders (NDDs), genes involved in RNA metabolic processing and regulation of gene expression have been shown to be enriched for de novo variants (DNVs) among probands with NDDs [1]. RNA processing (splicing, transport, localization, translation, and degradation) is critically important for brain development and function, as neurons are post-mitotic cells dependent on RNA expression, as well as spatiotemporal isoform specificity, for individual growth and functionality [2]. To successfully regulate RNA processing and protein synthesis, over 500 RNA-binding proteins (RBPs) in humans are abundantly and ubiquitously expressed, found primarily in the nucleus [3]. Although ubiquitous, there are tissue-specific changes in alternative splicing from RBPs resulting in cell-specific phenotypes [4]. As RBPs are necessary for many steps of neuronal RNA metabolism, there are multiple opportunities for dysfunction, which is highlighted by the range of neurological phenotypes resulting from variation in RBP-encoding genes, including neurodegenerative diseases, muscular atrophies, and various cancers. RBPs have also been implicated in NDDs, most notably *FMR1* in fragile X syndrome.

Here, we hypothesize that variation in gene families with related structure and function in the brain will result in subtypes of NDDs with related pathology. With over 80% of Mendelian disease-associated genes being part of gene families and/or having functionally redundant paralogs, this provides an opportunity to divide many NDD candidate genes into subgroups [5, 6]. In fact, it has recently been shown that DNVs are enriched among a subset of gene families in probands with NDDs [7]. This approach aids in understanding the biological impacts of variation in groups of genes, provides an opportunity for gene discovery, and allows for development of gene/protein family-specific therapeutics impacting a larger number of individuals than when targeting a single gene.

Focusing on RNA processing, we applied this gene family approach to identify gene families implicated in NDDs. Of the four gene families identified from previous meta-analyses of NDD exomes, the heterogeneous nuclear ribonucleoproteins (hnRNPs for proteins, *HNRNP*s for genes) stand out as strong NDD candidate genes that have yet to be fully investigated, as the fewest members of the family are associated with known disorders (Table 1). The hnRNPs are a large family of RBPs consisting of 33 core and minor members implicated in many steps of RNA processing. Several, primarily through changes in expression or localization, have been associated with neurodegenerative disorders, and, more recently, five (*HNRNPH1*, *HNRNPH2*, *HNRNPK*, *HNRNPR*, and *HNRNPU*) have been described as having DNVs among probands with NDDs (Table 2). We have also previously shown that two *HNRNP*s (*HNRNPU* and *SYNCRIP*) have a significant enrichment of DNVs among probands with NDDs, making them very strong candidate genes [1]. Supporting their role in NDDs as a gene family, it is known that hnRNPs function cooperatively and compensatorily, suggesting that disruption among them may result in similar phenotypic consequences. Thus, we hypothesize that there may be additional hnRNPs with shared structure and function that impact neurodevelopment, resulting in shared phenotypes. Overall, the hnRNPs are just one example of how multiple members of a gene family can be involved in related NDDs. Recent data from large-scale sequencing efforts suggest that other gene families (e.g., chromodomain DNA-binding helicase gene family) may benefit from similar coordinated investigations.

**Table 1:** Gene families involved in RNA processing reaching FDR significance in Coe et al. [1] and their role in disease

Gene family	Genes reaching FDR significance	# of NDD candidate genes in gene family
Chromodomain DNA-binding proteins	*CHD2*, *CHD3*, *CHD4*, *CHD7*, *CHD8*	6/10
BAF complex	*ARID1B*, *BCL11A*, *SMARCA2*, *SMARCA4*, *SMARCD1*	16/24
Heterogeneous nuclear ribonuclear proteins	*HNRNPU*, *SYNCRIP*, *HNRNPD*, *HNRNPK*	7/33
Lysine acetyltransferases	*CREBBP*, *DLG4*, *EP300*, *TAF1*	5/17

Gene families were defined by the HUGO Gene Nomenclature Consortium. At least three members of the gene family must reach FDR significance to be included. NDD candidate genes were determined by OMIM and a literature search. Coe et al. [1] *n* = 11,722 exomes

**Table 2:** Previous disease associations for genetic variation in *HNRNP* genes

Gene	Disorder	Type of variation
*HNRNPA1*	ALS/FTL [8] Multisystem proteinopathy [8, 9]	MIS MIS
*HNRNPA2/B1*	ALS/FTLD [8, 9] Multisystem proteinopathy [8, 9]	MIS MIS
*HNRNPA3*	ALS/FTLD [8, 9] Multisystem proteinopathy [8, 9]	MIS MIS
*HNRNPD/AUF1*	4q21 microdeletion/duplication/triplication syndrome [10]	CNVs
*HNRNPH1*	*HNRNPH1*-related syndromic ID [11]	MIS20/small CNVs
*HNRNPH2*	Bain-type ID [12]	MIS20
*HNRNPK*	AKS/Okamoto syndrome [13, 14] Kabuki syndrome [15–17]	LGD/MIS20/chromosomal deletions MIS
*HNRNPR*	*HNRNPR*-related disorder [18]	LGD/MIS20
*SYNCRIP/HNRNPQ*	6q proximal deletions [19]	Chromosomal deletion
*HNRNPP/FUS/TLS*	ALS/FTLD [3]	LGD/MIS
*HNRNPU*	1q43q44 microdeletion syndrome [20] *HNRNPU*-related disorder [20–23]	Chromosomal deletion LGD/MIS20/chromosomal duplication

*MIS* missense, *MIS20* missense variants with CADD scores ≥ 20, indicating they are in the top 1% of likely pathogenic variants, *CNVs* copy number variants, *LGD* likely gene disrupting, *ALS* amyotrophic lateral sclerosis, *FTLD* frontotemporal lobar degeneration, *ID* intellectual disability, *AKS* Au-Kline syndrome

Here, through international collaborations, protein, mutation and expression analyses, and an exhaustive literature search, we identify additional *HNRNP*s with pathogenic likely gene-disrupting (LGD) and severe missense single-nucleotide variants (SNVs), indels, or small copy number variants (CNVs) among probands with NDDs while utilizing novel methods of grouping genetic disorders by gene family instead of broader gene function. We compare findings from a total of 250 probands with NDDs with de novo or likely de novo SNVs/CNVs: 122 novel cases (118 SNVs or small indels and four chromosomal deletions) and 128 published probands (117 SNVs or small indels and 11 CNVs). Of these, 240 probands have variants occurring in 12 *HNRNP*s where three or more probands have been identified (termed NDD *HNRNP*s: *HNRNPAB*, *HNRNPD*, *HNRNPF*, *HNRNPH1*, *HNRNPH2*, *HNRNPH3*, *HNRNPK*, *HNRNPR*, *SYNCRIP*, *HNRNPU*, *HNRNPUL1*, and *HNRNPUL2*). We establish variation in a subset of the *HNRNP*s as a risk factor for NDDs and highlight the utility of a gene family-based approach to identify NDD-related disorders.

## Methods

### Identification of candidate NDD *HNRNP*s

#### Literature review to determine NDD candidate *HNRNP*s

**Fig. 1:**
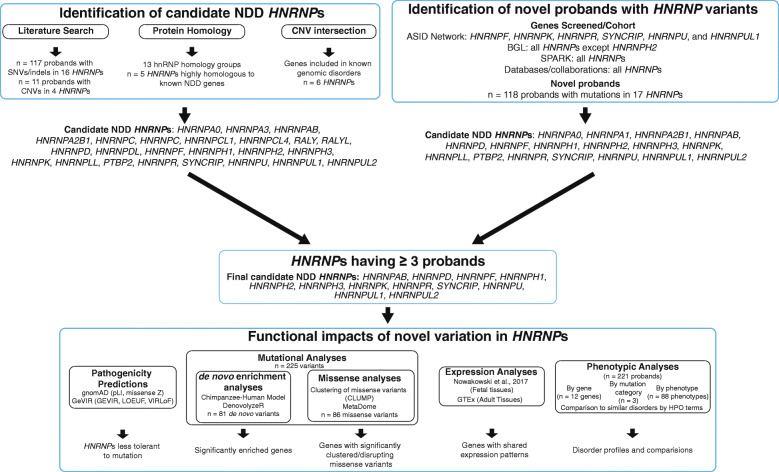
Study workflow. Candidate NDD *HNRNP*s were determined from the literature and publicly available information (such as amino acid sequences) and from identification of probands in our novel cohorts. The candidate NDD *HNRNP*s were finalized by considering only genes in which at least three probands were identified from published and/or novel sources. Functional impacts focused on these finalized NDD *HNRNP* candidates and included pathogenicity predictions (gnomAD and GEVIR), de novo enrichment analyses (using the Chimpanzee–Human [CH] model and denovolyzeR), missense analyses (using CLUMP and MetaDome), expression analyses of fetal cortex and adult tissues, and phenotypic analyses within *HNRNP*s, across *HNRNP*s, and in comparison to other similarly presenting disorders by HPO terms. CNV: copy number variant; pLI: loss-of-function intolerance; LGD: likely gene disrupting; NMD: nonsense mediated decay; HPO: human phenotype ontology

First, *HNRNP*s with published cases were identified in large exome or genome sequencing studies, as identified by denovo-db v1.6 or case reports found by searching PubMed with all known gene aliases (*n* = 128; Fig. 1, Additional File 1: Table S1) [11–18, 20–61]. Cohorts in denovo-db v1.6 include ASD samples (*n* = 5886: including the Simons Simplex collection [SSC, *n* = 2508] and MSSNG [*n* = 1625]), DD/ID samples (*n* = 1010), and epilepsy samples (*n* = 532, including the Epi4K Consortium [*n* = 264]). Samples were also identified from the Autism Sequencing Consortium (ASC, *n* = 8157) and the Epilepsy Genetics Initiative (*n* = 166). We also identified samples in the Deciphering Developmental Disorders (DDD) cohort, described below as we recalled the variants using our own analyses [39, 49, 62, 63]. In total, 51,616 published individuals were screened. Cases had to have a variant in an *HNRNP* to be included (i.e., not solely changes in gene expression as seen in some neurodegenerative phenotypes). Publications had to include at least one of the disorders under the HPO terms “abnormality of the nervous system physiology” (HP:0012638) and/or “abnormality of the nervous system morphology” (HP:0012639). This includes, but is not limited to, developmental delay/intellectual disability (DD/ID), seizures, and behavioral abnormalities such as autism spectrum disorder (ASD). Note that neurodegenerative phenotypes, such as ALS, were not included as part of this analysis. Clinical information was requested from authors when possible for larger sequencing studies. Duplicates were removed based on sample ID, sharing of multiple variants, and disclosure of duplicates in publications. When possible, samples were excluded if they had a likely causative variant in an NDD candidate gene, as determined by multiple publications [1, 58].

#### Protein homology analysis to identify additional candidate NDD *HNRNP*s

**Fig. 2:**
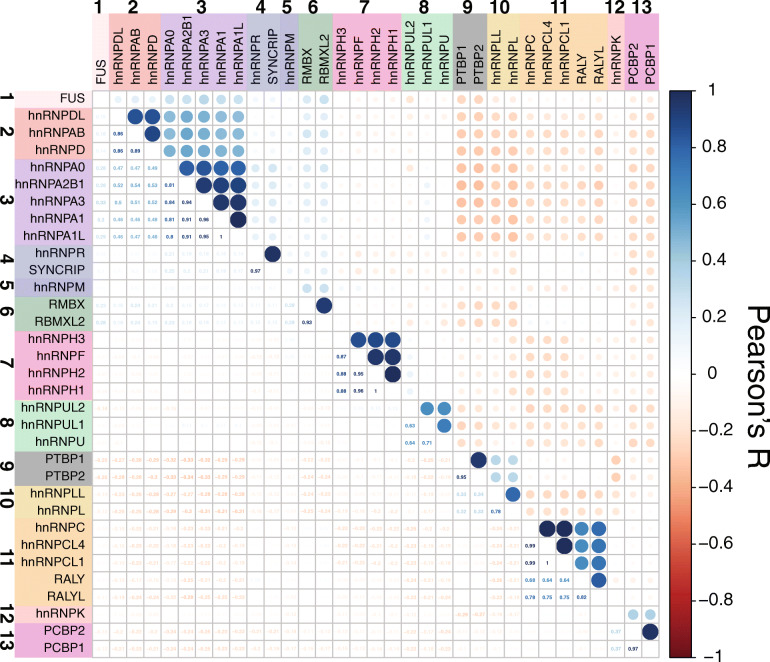
Protein similarity of hnRNPs. Correlation plot of hnRNPs by canonical amino acid sequence. Pearson correlation values are shown in the bottom half of the plot and are shown visually on the top half of the plot

To determine if there were a subset of hnRNPs with sequence similarity to known NDD hnRNPs, and thus potential candidate NDD genes, we utilized protein homology analysis (Fig. 2 and Additional File 2: Fig. S1). Protein homology was determined using Clustal Omega with canonical transcript sequences obtained from UniProt and clustering was performed in R (v.3.6.1) using the corrplot package (v.0.84) to identify shared protein homology.

### CNV intersection

In order to determine if any *HNRNP*s are dosage sensitive, we intersected the *HNRNP*s with a list of 58 genomic disorders based on previous CNV morbidity maps and the DECIPHER database (Table 3) [10, 13, 19, 20, 64–67]. From this, we identified 15 probands with CNVs spanning only an NDD *HNRNP* gene or neighboring genes not predicted to be haploinsufficient.

**Table 3:** Genomic disorders spanning NDD *HNRNP*s

Genomic disorder	***HNRNP***	Gene previously considered candidate for CNVs?	Shared phenotypes
5q35 deletions	*HNRNPH1*	No	DD/ID, characteristic facial features, overgrowth, microcephaly
1q43q44 deletions	*HNRNPU*	Yes	DD/ID, seizures, structural brain abnormalities, speech delay
9q21.32 deletions	*HNRNPK*	Yes	DD/ID, motor delay, speech delay, structural brain abnormalities, hypotonia, skeletal abnormalities, hand/feet abnormalities, cardiac abnormalities, genitourinary issues, dysmorphic features
6q proximal deletions	*SYNCRIP*	Yes	DD/ID, ASD, structural brain abnormalities, behavioral issues
4q21 microdeletion syndrome	*HNRNPD*	No	DD/ID, emotional/behavioral issues, speech delay
1p36 monosomy	*HNRNPR*	No	DD/ID, skeletal abnormalities, genitourinary issues, seizures, structural brain abnormalities

*CNV* copy number variant, *DD/ID* developmental delay/intellectual disability, *ASD* autism spectrum disorder

### Identification of novel probands with *HNRNP* variants

**Fig. 3:**
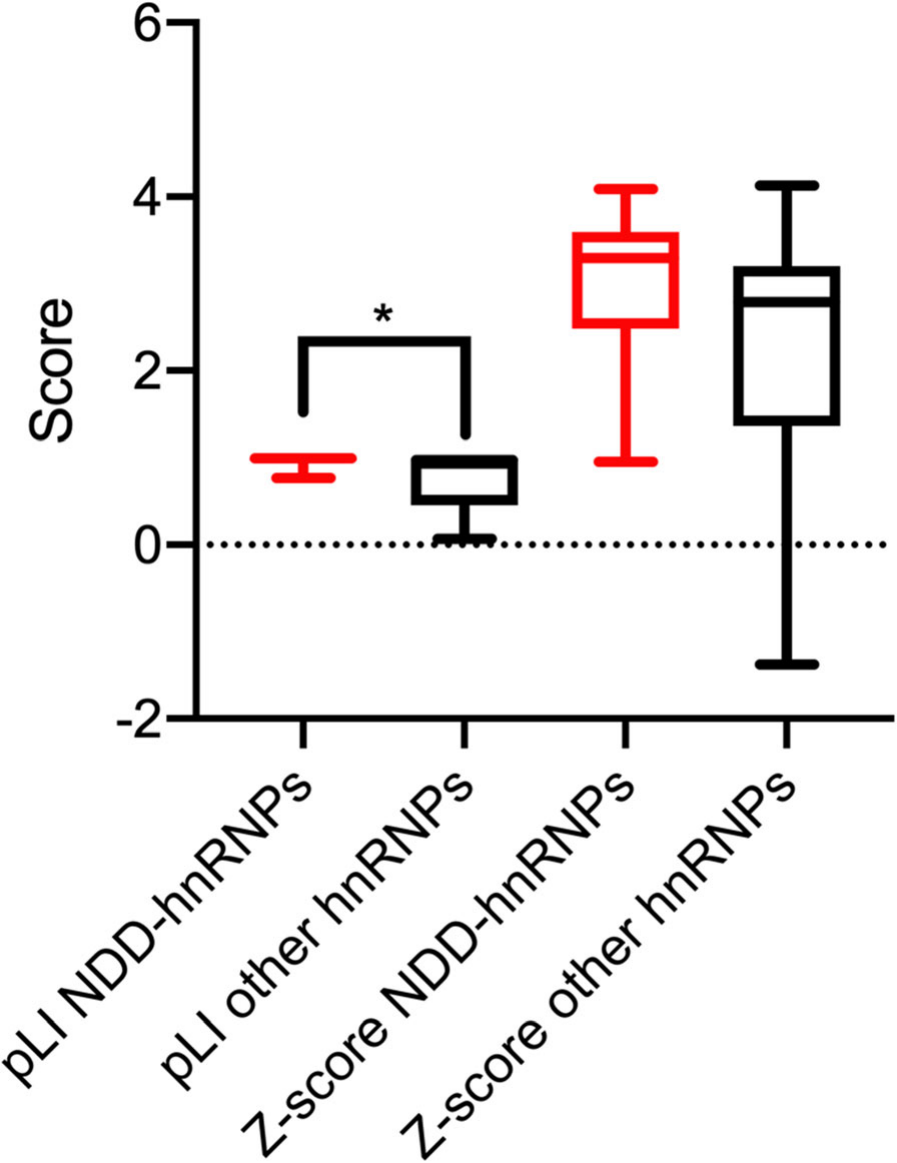
Pathogenicity assessment of variation in hnRNPs. pLI and *Z*-scores were obtained from gnomAD. pLI scores are significantly higher among NDD hnRNPs (*n* = 12) compared to non-NDD hnRNPs (*n* = 20), suggesting LGD variants are more likely to be damaging. *Z*-scores trend towards being significantly higher for NDD hnRNPs, suggesting severe missense variants are likely to be damaging. *T*-test with Welch’s correction. *p < 0.05. pLI: loss-of-function intolerance

Probands were collected from multiple cohorts (*n* = 32,359 probands) (Additional File 1: Table S1). We also queried ClinVar and DECIPHER databases. We identified 122 probands with SNVs, indels, or CNVs impacting 14 *HNRNP*s (Fig. 3, Additional File 1: Tables S7, S10 and S11).

Families where recontact was possible were invited to participate in a remote comprehensive clinical workup that included diagnostic evaluation, medical history, and neuropsychological assessment. All experiments carried out on these individuals were in accordance with the ethical standards of the responsible committee on human experimentation (institutional and national), and proper informed consent was obtained for sequencing, recontact for inheritance testing, and phenotypic workup. When physicians or families could not be contacted, deidentified clinical information was used. Probands were excluded if they reportedly had (1) a causative variant in another NDD-related gene, (2) a known inherited variant in an *HNRNP* (other than mosaic), (3) a variant observed in gnomAD, (4) a missense variant with a CADD (v1.6, GRCh38) score < 20, (5) consanguineous parents (which was based on clinical history), and/or were (6) born prematurely (prior to 37 weeks), or (7) too young to assess for DD (< 1 year). However, individuals excluded for our *HNRNP*s cohort were still considered in the total number of individuals for statistical analyses. For probands from large sequencing studies, these criteria were not always available; therefore, a small number of those not meeting our criteria may be included due to limited information.

### Cohorts

#### ASID Network

For our Autism Spectrum/Intellectual Disability (ASID) network (*n* = 16,294 individuals), six *HNRNP*s were previously assessed using targeted sequencing with single-molecule molecular inversion probes: *HNRNPF*, *HNRNPK*, *HNRNPR*, *SYNCRIP*, *HNRNPU*, and *HNRNPUL1*, as described by Stessman et al., identifying 14 probands (seven with DNVs) included in the current study [34, 68]. All variants were validated using Sanger sequencing, although not all had parental DNA available for inheritance testing. Information for a subset of these probands are available in denovo-db v1.6.

#### BGL

From Baylor Genetics Laboratory (BGL; *n* = 9536, including 1744 trios), 34 novel probands are included in the current study (four with DNVs) and four previously published probands with DNVs (for a total of 38 probands) [23]. These probands are all from clinical exome sequencing samples broken down into “neurologic” phenotypes (*n* = 2364, including 429 trios; developmental delay [DD]/intellectual disability [ID], speech delay, and autism spectrum disorder [ASD]) and “neurologic plus other organ systems” (*n* = 7172, including 1315 trios). Exome sequencing was performed as described previously, and DNVs were validated by Sanger sequencing [69]. All *HNRNP*s were queried in the BGL data except *HNRNPH2*. Phenotypic information was obtained from BGL clinical indications for sequencing and/or from reporting physicians when available.

#### SPARK Consortium

From the SPARK Consortium, with individuals ascertained for ASD diagnoses, nine probands with DNVs are included in the current study, as well as an additional published proband (for a total of 10 probands) [42]. Probands identified by the SPARK Consortium were sequenced as previously described [42]. SPARK Consortium probands were used in all statistical analyses, although complete phenotypic information was not always available.

#### Deciphering Developmental Disorders (DDD13K)

Data for 9860 trios from the DDD cohort were obtained and reanalyzed [62]. We applied FreeBayes (version v1.1.0–3-g961e5f3) and GATK (version 3.7) for SNV/indel calling independently in 9308 DDD families where both parents were available (with 28,476 samples including 9860 probands) [70, 71]. BCFtools (version 1.3.1) was used for left-align and normalization post calling [72]. For quality control, we filtered for read depth (DP > 9) for all family members and filtered allele balance (AB > 0.25) and PHRED genotype quality (GQ > 20) filters in probands. Candidate de novo SNVs/indels were only observed in probands but not in parents and were required to be identified by both FreeBayes and GATK. Variants were then annotated using CADD (v1.6, GRCh38).

#### International collaborations

GeneMatcher and GeneDx were utilized to identify 24 individuals, 21 of whom have DNVs [73]. GeneMatcher requests were made for each *HNRNP* identified in an NDD cluster (Fig. 2). Probands were sequenced at varying locations, including several by GeneDx, whose protocol was described previously. Deidentified clinical information was obtained from physicians or families were invited for remote clinical workup. These probands were not used in de novo statistical calculations but were considered among phenotypic analyses.

Four probands with DNVs were obtained from the Radboud University Medical Center (RUMC) in the Netherlands. These probands were not used in de novo statistical calculations but were considered among phenotypic analyses.

#### Databases

Large databases were also used to identify novel probands. Each hnRNP-encoding gene was queried in MyGene2 (https://mygene2.org/MyGene2/), a database generated by the University of Washington Center for Mendelian Genomics, identifying two probands (one proband was identified via both MyGene2 and GeneMatcher) [74]. MyGene2 probands were not used for de novo statistical calculations but were considered for phenotypic analyses.

DECIPHER (*n* > 33,000 individuals; https://decipher.sanger.ac.uk/) and ClinVar (*n* = 874,088; https://www-ncbi-nlm-nih-gov.offcampus.lib.washington.edu/clinvar/) were queried and only those with phenotypic information available were included in this study, adding 12 (10 with DNVs) and 19 (seven with DNVs) new probands, respectively. Probands were considered novel if they had not been previously published. DECIPHER and ClinVar probands were included in phenotypic analyses but not de novo statistics.

### Functional impacts of novel variation in *HNRNP* genes

#### Pathogenicity predictions, de novo enrichment, and missense analyses

To further characterize the types of variation among NDD *HNRNP*s, we compared the probability of pathogenicity of LGD and missense variants using gnomAD (loss-of-function intolerance [pLI] and missense Z-scores; https://gnomad.broadinstitute.org/) and Gene Variation Intolerance Ranking (GeVIR; http://www.gevirank.org/) scores and percentiles, which include loss-of-function observed/expected upper bound fraction (LOEUF) metrics, of NDD *HNRNP*s to non-NDD *HNRNP*s using a one-way *t*-test in GraphPad Prism (v.8.3.0) (Fig. 3 and Additional File 2: Fig. S3, Additional File 1: Table S2) [75].

**Fig. 4:**
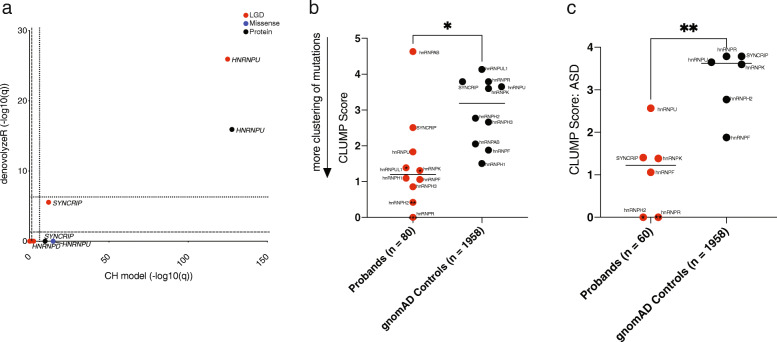
De novo enrichment and clustering of missense variation analyses of NDD hnRNPs. **a** De novo variation was assessed for NDD *HNRNP*s using two statistical models: the CH model and denovolyzeR. Right/above the dotted line indicates the gene achieves exome-wide significance (*q* < 4.24 × 10^− 7^) while right/above the dashed line indicates the gene reaches nominal significance (*q* < 0.05). *HNRNPU* reaches exome-wide significance for all protein-impacting variants (Protein) and LGD variants, with severe missense variants reaching significance by only the CH model. *SYNCRIP* reaches exome-wide significance for LGD variants and all protein-impacting variants by the CH model alone. *HNRNPD* reaches nominal significance by the CH model. *P* values are FDR corrected with the number of genes (*n* = 18,946 for CH model and *n* = 19,618 for denovolyzeR) with three tests per gene (LGD, missense, and all protein changes) and two tests (CH model and denovolyzeR) per mutation type. Only cohorts with known de novo status were included, as listed in Tables S1 and S7. Statistics can be seen in Table S6. **b** Analysis of clustered missense variants. Clustering of missense variants was analyzed using CLUMP; scores are shown in Table S6 (paired *t*-test). Compared to the non-neuropsychiatric subset of gnomAD (*n* = 114,704, 1958 missense variants), the CLUMP score for NDD hnRNPs (red) among probands is significantly lower than controls in gnomAD (black), indicating more clustering of mutations (shown by arrow). Note that only genes with variants in the current cohort could undergo this analysis. hnRNPH2, hnRNPK, hnRNPR, and hnRNPUL1 each have independent significant clumping compared to gnomAD controls. **c** CLUMP scores for missense variants in probands with ASD (*n* = 60). HnRNPH2, hnRNPK, hnRNPR, and hnRNPUL1 reach significance independently. *p < 0.05, **p < 0.01, ***p < 0.001. LGD: likely gene disrupting; Missense: severe missense (CADD ≥ 20); Protein: all protein-affecting variants

For de novo enrichment analyses, 33,688 probands with trio sequencing (*n* = 36,460 for *HNRNPH2* and *n* = 36,814 for *HNRNPU*) were used (Additional File 1: Table S6). Only studies with a clear sample size were used to estimate de novo burden (i.e., *n* > 1, cohort total sizes included in publication). Only genes determined as NDD *HNRNP*s were analyzed. In total, 78 *HNRNP* variants were analyzed (Additional File 1: Table S7). Two models, the chimpanzee–human divergence (CH) model and denovolyzeR, were used to assess the excess of de novo LGD and missense variants as previously described (Fig. 4a) [1, 34, 76, 77]. Briefly, the CH model utilizes locus-specific transition/transversion/indel rates and chimpanzee–human coding sequence divergences to estimate the expected number of de novo mutations, while the denovolyzeR utilizes trinucleotide context, exome depth, and divergence between macaque and human over a ± 1 megabase-pair window and includes known mutational biases like CpG hotspots. Each model was corrected for the number of genes included in the model (*n* = 18,946 for the CH model and *n* = 19,618 for denovolyzeR) as well as three mutation-type tests per gene and two tests per mutation type (FWER for CH model: *q* < 4.4 × 10^− 7^, FWER for denovolyzeR *q* < 4.24 × 10^− 7^).

**Fig. 5:**
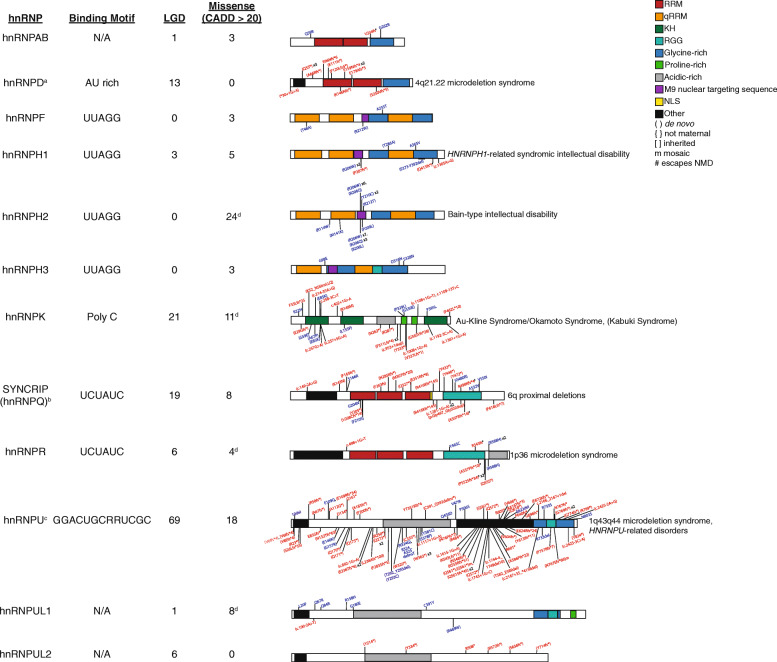
hnRNP proband variants. Protein structure, known binding motif, number of probands by mutation type, location of variants in each protein, and known associated disorders of NDD hnRNPs are shown. Novel cases are above the protein with published cases below. Red indicates LGD variants and blue represents severe missense variants. RRM: RNA recognition motif; qRRM: quasi-RNA recognition motif; KH: K-homology domain; RGG: Arginine-glycine rich (RGG) box; NLS: nuclear localization sequence. Further details of each variant are shown in Table S7. Adapted from Geuens et al. [83]. **a** Gene reaches exome-wide significance for all protein-impacting variants by CH model. **b** Gene reaches exome-wide significance for all protein-impacting variants and LGD variants by CH model. **c** Gene reaches exome-wide significance for all protein-impacting variants and LGD variants by CH model and denovolyzeR. **d** Gene reaches significance for missense variant clustering

Missense variants were analyzed using two methods: CLUMP (CLUstering by Mutation Position) analysis and MetaDome assessment [78–80]. Only genes determined NDD *HNRNP*s were analyzed. CLUMP assesses the clustering of missense mutations and was performed as previously described, but here using the gnomAD non-neuropsychiatric group as controls (gnomAD *n* = 114,704; 1958 missense variants; current cohort missense *n* = 80 variants) (Fig. 4b, Additional File 1:Table S6) [80]. All calculations were performed using R (v.4.0.0) or python (v.3.7.7). Missense variants were also assessed for clustering by phenotype. Mutations were queried in MetaDome to assess for protein domain disruptions (Fig. 5) [78]. LGD variants were also assessed for susceptibility to nonsense mediated decay (NMD) using NMDEscPredictor (https://nmdprediction.shinyapps.io/nmdescpredictor/) and splicing impact using MaxEntScan (Fig. 5, Additional File 1: Table S7) [81, 82].

#### GTEx and single-cell expression analyses

**Fig. 6:**
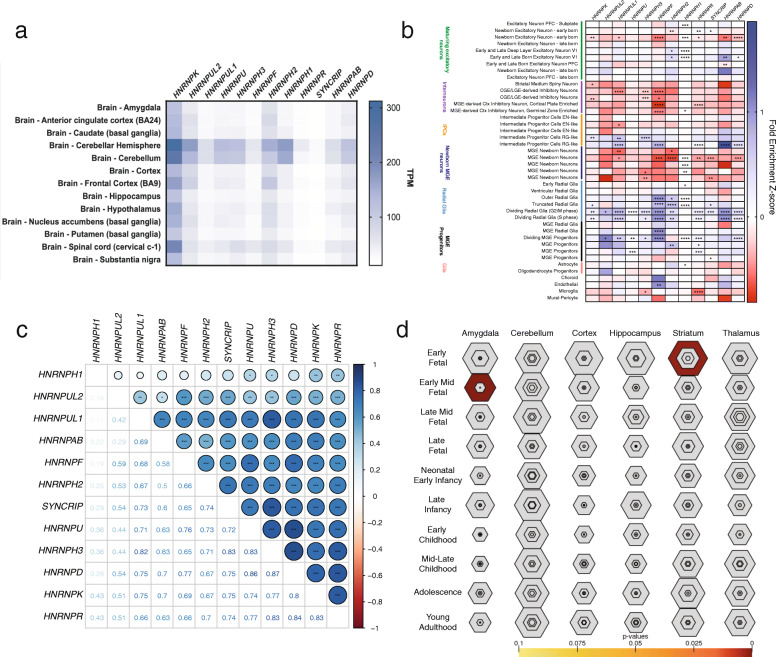
*HNRNP* expression in adult and developing fetal cortex tissues. **a** Heatmap showing transcript-level expression values for NDD hnRNPs for adult brain tissues in GTEx. All tissues are shown in Fig. S4, and *p* values among individual *HNRNP*s are shown in Table S3. **b** Heatmap showing fold change of expression of each NDD *HNRNP* among 48 different cell types in the developing fetal cortex. Blue indicates increase in fold change of expression and red indicates decreased expression, as determined by *Z*-scores. NDD *HNRNP*s have higher fold expression change in MGE progenitors, radial glia, and excitatory neurons, while depleted in inhibitory neurons. All *HNRNP*s are shown in Fig. S3. Significance indicates enrichment in particular cell types by Wilcoxon ranked sum test with Bonferroni correction based on number of cell types. *P* values and fold change for scRNA data from developing human cortex can be seen in Tables S4 and S5. **c** Correlation plot of developing fetal cortex gene expression. Pearson correlation *R* values are shown in the bottom half of the plot, which are visually in the top half of the plot. *P* values were corrected by number of genes [23] and number of cell types [48]. *HNRNP*s in the same homology group tend to have more correlated expression. **d** Specific brain region enrichment as determined by SEA, showing enrichment of expression of the NDD *HNRNP*s in the early fetal striatum and early-mid fetal amygdala. *p < 0.05; **p < 0.01; ***p < 0.001, ****p < 0.0001

Transcript-level expression from adult tissues obtained from GTEx (https://gtexportal.org/) for each gene was compared using *t*-tests between NDD and non-NDD *HNRNP*s as well as by two-way ANOVA for comparing between individual genes (Fig. 6a, Additional File 2: Figs. S3C,D, Additional File 1: Table S3). We used previously described single-cell RNA (scRNA) sequencing data generated from 48 individuals to assess gene expression during fetal human cortical development, which is available from the UCSC cell browser: https://cells.ucsc.edu/ [84]. To determine cell enrichment, data were tested using a Wilcoxon ranked sum test followed by Bonferroni correction in R, as previously described [84]. Student *t* tests and two-way ANOVA among all *HNRNP*s, between NDD and non-NDD *HNRNP*s, or *HNRNP*s and genes associated with similarly presenting disorders determined the difference in fold-enrichment using GraphPad Prism (Figs. 6b,c and 8b,c, Additional File 2: Figs. S3A,B, Additional File 1: Tables S4–5). Correlations among scRNA sequencing data were performed in R (v.3.6.1) using the corrplot package (v.0.84). One-way *t*-tests were performed between each NDD *HNRNP* group (as in Fig. 2) using GraphPad Prism. *HNRNP*s were also entered into Specific Expression Analyses (SEA) to determine enrichment of expression across brain regions (Fig. 6d) [85]. Expression among these tissues was compared using Fisher’s exact tests and followed by Benjamini-Hochberg correction.

#### Phenotypic analyses

Phenotypes were defined by Human Phenotype Ontology (HPO) terms when possible. Those that occurred in ≥ 20% of probands in at least one *HNRNP* cohort were considered characteristic for the disorder and used for statistical analyses. Traits occurring less than that were considered not likely to be syndromic. This resulted in a total of 88 phenotypes. For quantitative measures (e.g., head circumference), qualitative terms were used if provided by a clinician (e.g., microcephalic) or determined based on top/bottom 3rd percentile.

**Fig. 7:**
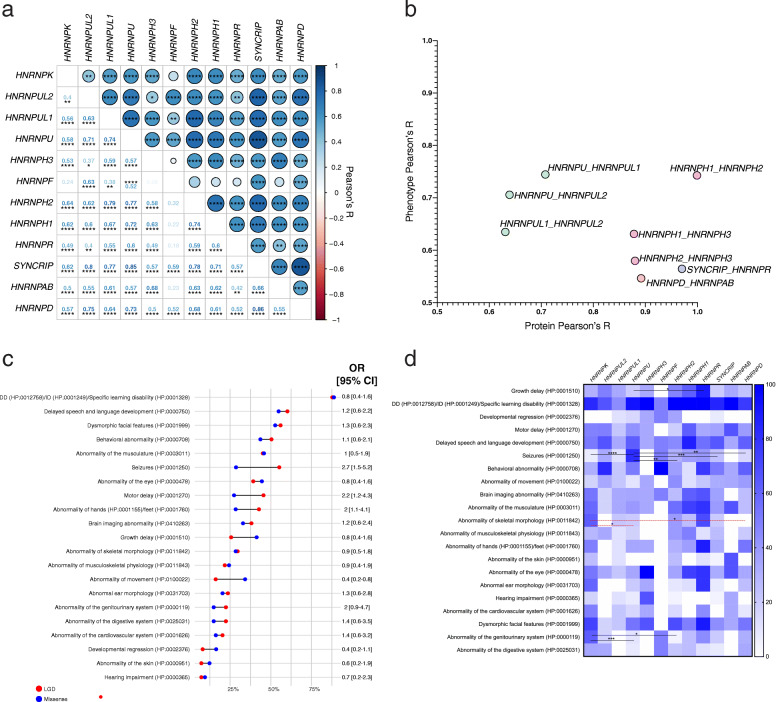
Phenotypic information of 189–221 hnRNP-variation probands. **a** Correlation matrix of phenotypes across hnRNP probands. Genes are in order of protein similarity as determined by Clustal Omega and canonical protein sequences as in Fig. 1. Phenotypes correlate across all *HNRNP*s, except *HNRNPF* due to sample size. Size and shade of circle represent correlation coefficients, which are shown on bottom half of matrix. Correlations for LGD and missense variants separately are in Fig. S4. *P* values, which are corrected by number of genes [23] and phenotypes (88, occurring in at least 20% of any *HNRNP* group) can be seen in Table S9. **b** Plot comparing protein and phenotype correlations that are over Pearson’s *R* = 0.5. Colors are the same as in Fig. 2 protein groups. Those with more similar protein sequences tend to be more phenotypically similar. **c** Plot of phenotypes of all probands by mutation type. Individual *HNRNP*s can be seen in Fig. S5. **d** Heatmap indicating percent of probands with phenotype. Sample sizes can be seen in Table S7 and range from *n* = 2 (*HNRNPF*) to *n* = 83 (*HNRNPU*). Lines indicate significant differences as determined by pairwise Fisher’s exact tests with Bonferroni correction based on 12 genes, 88 phenotypes, and three mutational categories. Red dashed lines indicate significance with only LGD variants. Raw *p* values can be seen in Tables S8. *p < 0.05, **p < 0.01, ***p < 0.001, ****p < 0.0001. LGD: likely gene disrupting; MIS: severe missense (CADD ≥ 20)

Phenotypic information was compared across *HNRNP* cohorts using RVAideMemoire (version 0.9–73) pairwise Fisher’s exact tests with Bonferroni correction considering number of genes (*n* = 12 for all NDD *HNRNP*s, *n* = 9 for LGD NDD *HNRNP*s, *n* = 10 for missense NDD *HNRNP*s), number of phenotypes (*n* = 88), and number of mutation categories (*n* = 3: all mutations, LGD, and missense) tested, as well as across mutation types (LGD vs. missense) within *HNRNP* cohorts using Fisher’s exact tests (Fig. 7, Additional File 1: Tables S8–9). All calculations were performed using GraphPad Prism or R. Phenotypes were compared to other known disorders by characteristic HPO terms using PhenPath (http://phenpath.biocomp.unibo.it/phenpath/), which returns disorders and genes with shared HPO terms [86].

## Results

### Twelve *HNRNP*s are NDD candidates based on literature search, protein homology, CNV intersection, and identification of new probands

In order to identify NDD candidate *HNRNP*s, we initially considered cases with DNVs in the literature. This resulted in 16 genes with at least one published case as potential NDD candidate genes: *HNRNPA0, HNRNPA3, HNRNPAB*, *HNRNPA2B1*, *HNRNPC*, *HNRNPD*, *HNRNPF*, *HNRNPH1*, *HNRNPH2*, *PTBP2*, *HNRNPK*, *HNRNPLL*, *SYNCRIP*, *HNRNPR*, *HNRNPU*, and *HNRNPUL1* (*n* = 117 probands; Fig. 1, Additional File 1: Tables S1, S7, S8, and S11)*.*

To identify other genes in the family that may not have published cases, we considered the extent of shared protein homology among hnRNPs (Fig. 2 and Additional File 2: Fig. S1). Among 23 core hnRNPs and 10 minor hnRNPs, we distinguished 13 protein groups based on protein homology clustering: (1) FUS (Fused in sarcoma, also known as hnRNPP), (2) hnRNPD-related proteins, (3) hnRNPA-related proteins, (4) hnRNPR/SYNCRIP, (5) hnRNPM, (6) RMBX (RNA-binding motif protein X-linked, also known as hnRNPG)-related proteins, (7) hnRNPH/F-related proteins, (8) hnRNPU-related proteins, (9) PTBP (also known as hnRNPI)-related proteins, (10) hnRNPL-related proteins, (11) hnRNPC-related proteins, (12) hnRNPK, and (13) PCBP (also known as hnRNPE)-related proteins. From this, we identified seven additional hnRNPs that, while they do not have published cases with NDDs, cluster with our other candidate hnRNPs: hnRNPCL1, hnRNPCL4, RALY, RALYL, hnRNPH3*,* hnRNPDL, and hnRNPUL2.

We determined if any *HNRNP*s are dosage sensitive by intersecting the *HNRNP*s with a list of 58 genomic disorders based on previous CNV morbidity maps and the DECIPHER database (Table 3) [10, 13, 19, 20, 64–67]. Of the 33 *HNRNP*s, six are in regions of known genomic CNV disorders: *HNRNPD*, *HNRNPH1*, *HNRNPK*, *HNRNPR*, *SYNCRIP*, and *HNRNPU*, further supporting their likely role in NDDs. This analysis did not identify any new candidate NDD *HNRNP*s but did provide additional evidence for their roles in NDD pathogenicity.

Genes were also considered as candidate NDD *HNRNP*s if we identified variants in our novel cohorts. This resulted in 17 genes with at least one novel case as potential NDD candidate genes: *HNRNPA0, HNRNPA1*, *HNRNPA2B1*, *HNRNPAB*, *HNRNPD*, *HNRNPF*, *HNRNPH1*, *HNRNPH2*, *HNRNPH3*, *HNRNPK*, *HNRNPLL*, *PTBP2*, *SYNCRIP*, *HNRNPR*, *HNRNPU*, *HNRNPUL1*, and *HNRNPUL2* (*n* = 118 probands; Fig. 1, Additional File 1: Tables S1, S7, S8, and S11)*.*

Finally, NDD *HNRNP*s were finalizing by identification of at least three probands in our combined literature and novel cohorts. This resulted in the following being considered NDD *HNRNP*s: *HNRNPAB*, *HNRNPD*, *HNRNPF*, *HNRNPH1*, *HNRNPH2*, *HNRNPH3*, *HNRNPK*, *HNRNPR*, *SYNCRIP*, *HNRNPU*, *HNRNPUL1*, and *HNRNPUL2*.

### NDD *HNRNP*s are more sensitive to mutation than non-NDD *HNRNP*s

We considered each gene’s tolerance to mutation by assessing gnomAD metrics pLI and missense *Z*-scores as well as GeVIR scores (Fig. 3 and Additional File 2: Fig. S2, Additional File 1: Table S2) [75, 87]. As a group, pLI scores are significantly higher among NDD *HNRNP*s compared to non-NDD *HNRNP*s (*p* = 0.03, one-way Student’s *t* test). Similarly, NDD *HNRNP*s’ missense *Z*-scores trend higher than non-NDD *HNRNP*s; GeVIR scores show the same pattern. Specifically, NDD *HNRNP*s have significantly higher LOEUF autosomal dominant scores (*p* = 0.03, one-way Student’s *t* test) and Variation Intolerant Region Loss-of-Function (VIRLoF) scores, which takes both missense and LGD variants into account (*p* = 0.04, one-way Student’s *t* test). GeVIR autosomal recessive scores show a similar pattern for LOEUF scores (*p* = 0.04, one-way Student’s *t* test), but as all of our variants are heterozygous, this information was not utilized in our study. This suggests that LGD and severe missense variants are more likely to be damaging among NDD *HNRNP*s compared to the non-NDD *HNRNP* family members.

### Enrichment of de novo *HNRNP* variants

In total, 225 probands with SNVs or indels in NDD *HNRNP*s were identified from 83,975 individuals. Inheritance was determined for 73.8% of NDD *HNRNP* SNV and indel variants (Table 4, Fig. 3, and Additional File 1: Table S7). Of variants with known inheritance, DNVs account for 98.8% of variants, with one mosaic variant inherited from the unaffected father, who also carries the variant mosaically (Proband 124, *HNRNPU* proband 1) [26]. LGD variants represent 61.8% (*n* = 139/225 variants, inheritance known for 82.7%; 99.1% de novo) of the NDD *HNRNP* cohort, while severe missense variants (MIS20: CADD score ≥ 20, being in the top 1% of predicted pathogenic mutations and MIS30: CADD ≥ 30 top 0.1% of predicted pathogenic variants) account for 38.2% of variants (*n* = 86/225 variants, inheritance known for 59.3%; 98% de novo; MIS20, MIS30, and those without CADD scores available were included). Twelve of the 16 small CNVs have known inheritance status, and all are de novo (Additional File 1: Table S12).

**Table 4:** Inheritance of 225 SNVs and indels in NDD *HNRNPs*

Variant type	De novo % of variant type (***n***)	Inherited % of variant type (***n***)	Unknown % of variant type (***n***)	Total % of all variants (***n***)
**LGD**	82 (114/139)	0.7 (1/139)	17.3 (24/139)	61.8 (139/225)
**All MIS**	58.1 (50/86)	1.2 (1/86)	40.7 (35/86)	38.2 (86/225)
**MIS—no CADD score**	50 (4/8)	0 (0/8)	50 (4/8)	3.6 (8/225)
**MIS20**	57.5 (42/73)	1.4 (1/73)	41.1 (30/73)	32.4 (73/225)
**MIS30**	80 (4/5)	0 (0/5)	28.6 (2/5)	2.7 (6/225)
**All variants**	73.2 (164/225)	0.9 (2/225)	25.3 (56/225)	100 (225/225)

*LGD* likely gene disruptive, *MIS* missense, *MIS20* CADD score ≥ 20, *MIS30* CADD score ≥ 30. NDD *HNRNP*s include *HNRNPAB*, *HNRNPD*, *HNRNPF*, *HNRNPH1*, *HNRNPH2*, *HNRNPH3*, *HNRNPK*, *SYNCRIP*, *HNRNPR*, *HNRNPU*, *HNRNPUL1*, and *HNRNPUL2*. Variants identified in non-NDD *HNRNP*s are in Table S7

In order to identify genes with a significant excess of DNVs, we examined 41,779 parent–child trios from 13,437 families with DD/ID, 20,542 families with ASD, 1421 families with epilepsy, and 6379 families from clinical exome testing. We applied two statistical models, denovolyzeR and the CH model, to genes that had DNVs from cohorts with known sample size (*n* = 78 variants, Fig. 4a, Additional File 1: Table S6) [76, 77]. We find that only *HNRNPU* achieves exome-wide significance (*p* < 4.24 × 10^− 7^) for de novo LGD and all protein-impacting variants by both models. *SYNCRIP*, for all protein-impacting variants and LGD variants, reaches exome-wide significance only by the CH model, while all protein-impacting variants of *HNRNPD* reach nominal significance (*p* < 0.05) by the CH model. Only *HNRNPU* reaches exome-wide significance for an excess of severe de novo missense variation after multiple testing correction (FWER, *p* < 4.24 × 10^− 7^), and only by the CH model. Based on these DNV models, only *HNRNPU*, *SYNCRIP*, and *HNRNPD* reach significance for increased number of variants than one would expect.

### Missense mutation analyses

As pathogenic missense variants are known to cluster for some proteins, such as hnRNPH2 in the M9 nuclear targeting signal, we assessed whether there was any additional evidence of missense variant clustering across hnRNPs [12]. We examined all MIS20 and MIS30 variants in our cohort (*n* = 80) and compared them to the non-neuropsychiatric subset in gnomAD using CLUMP (*n* = 1958) (Fig. 4b, Additional File 1: Table S6) [80]. In order to control for platform differences, variants were only included if exon coverage was at least 20-fold in gnomAD (the first two exons of *HNRNPUL1* were excluded). The CLUMP scores for NDD hnRNP missense variants are significantly lower than those of controls from gnomAD (*p* = 0.03, paired *t*-test) suggesting clustering of variants. hnRNPH2, hnRNPK, hnRNPR, and hnRNPUL1 independently have significantly more clustering of missense variants than controls (*p* < 0.05 for hnRNPK and hnRNPUL1, *p* < 0.01 for hnRNPH2 and hnRNPR). Thus, for multiple hnRNPs, clustering of missense variants likely contributes to pathogenicity, potentially impacting particular protein domains. Notably, this clustering was more significant among particular phenotypes, including ASD (*p* = 0.005, *HNRNPR* alone *p* = 0.007). In hnRNPH2, missense variants cluster significantly among probands with growth delay (*p* = 0.01), motor delay (*p* = 0.001), speech delay (*p* = 0.006), microcephaly (*p* = 0.01), hypotonia (0.003), seizures (*p* = 0.01), and cardiac abnormalities (*p* = 0.02) compared to controls. Cardiac abnormalities also have a clustering of missense variants among *HNRNPK* probands (*p* = 0.004). Variants cluster among probands with hypospadias, joint hypermobility, and scoliosis among *HNRNPH1* probands (*p* = 0.04 for each). In addition to ASD, variants in probands with speech delay or microcephaly and *HNRNPR* variants are clustered (*p* = 0.005 and *p* = 0.001, respectively). We also applied MetaDome to identify such domains and as another predictor of pathogenicity. There was no significant difference between variants in known domains and unknown domains. Other than the M9 nuclear localization signal, there was clustering of missense variants in the RNA-binding domain of hnRNPH2. In hnRNPK, 70% of missense variants (*n* = 7/10) cluster in the KH domains, which are important in RNA binding and recognition. Almost half (42.9%, *n* = 3/7) of missense variants in SYNCRIP cluster in its RNA recognition motif while de novo missense mutations in hnRNPU cluster in the SPRY domain and AAA domain (*n* = 3/16 each). Twenty-five percent (2/8) of missense variants in hnRNPUL1 occur in the SAP domain, important for RNA and DNA binding. Thus, RNA recognition and binding are likely responsible for many of the missense variants observed in the hnRNPs.

### *HNRNP* brain expression analyses

In order to understand the pathogenesis of variation in *HNRNP*s, we explored the expression of the genes in both adult tissues and the developing human cerebral cortex [84]. GTEx analysis of adult tissues shows that the *HNRNP*s as a whole (*n* = 33) are expressed across all brain regions, as well as ubiquitously across tissues, with the exception of *HNRNPCL1*, *RALYL* (*HNRNPCL3*), *HNRNPCL4*, and *RBMXL2,* which broadly show low levels of expression (Fig. 6a and Additional File 2: Figs. S3C,D). Significant changes in expression are seen based on individual genes and by tissue type (*p* < 0.0001 for both by two-way ANOVA), with highest expression levels in the brain seen typically in the cerebellum. Between NDD and non-NDD *HNRNP*s, no significant differences are observed (Additional File 2: Fig. S3). The general high expression of these genes may contribute to phenotypes observed.

scRNA sequencing from the developing cortex confirms widespread expression across brain cells, especially neuronal and neuronal progenitor cell types, suggesting that these genes could play a role in neuronal differentiation (Fig. 5b and Additional File 2: Figs. S4C,D) [84]. We find that *HNRNP* expression is enriched among radial glia, which act as neural stem cells, but decreased among immature inhibitory and excitatory neurons, although it does vary by *HNRNP* (*p* < 0.05 to *p* < 0.0001, Wilcoxon ranked sum test, Bonferroni correction; Fig. 6a and Additional File 2: S3C, Additional File 1: Table S4). While significant differences are seen by two-way ANOVA between individual *HNRNP*s, particularly for *PCBP1* (*HNRNPI)* and *HNRNPCL3/RALYL*, no significant differences are observed between all NDD and non-NDD *HNRNP*s (Additional File 2: Figs. S3A,B, Additional File 1: Table S5). It is plausible that there are differences between NDD and non-NDD *HNRNP* expression that contribute to the development of NDD phenotypes when perturbed.

Specifically, NDD *HNRNP*s are consistently significantly enriched among dividing radial glia in G2/M phase (all but *HNRNPH1*; *p* < 0.05 to *p* < 0.0001, Wilcoxon ranked sum test, Bonferroni correction), dividing radial glial cells during S phase (*HNRNPK*, *HNRNPUL1*, *HNRNPH3*, *HNRNPF*, *HNRNPH2*, *HNRNPR*, *HNRNPAB*, and *HNRNPD*; *p* < 0.05 to *p* < 0.0001, Wilcoxon ranked sum test, Bonferroni correction), and in the medial ganglionic eminences (MGE), which give rise to major populations of inhibitory neurons of the cortex (*HNRNPUL2*, *HNRNPUL1*, *HNRNPU*, *HNRNPH3*, *HNRNPF*, *HNRNPH1*, *HNRNPR*, and *HNRNPD*, *p* < 0.05 to *p* < 0.0001, Wilcoxon ranked sum test, Bonferroni correction), while depleted in newborn neurons in the MGE (*HNRNPUL1*, *HNRNPH3*, *HNRNPF*, *HNRNPH2*, *HNRNPH1*, *HNRNPR*, *SYNCRIP*, and *HNRNPD*; *p* < 0.05 to *p* < 0.0001, Wilcoxon ranked sum test, Bonferroni correction) and newborn excitatory neurons (*HNRNPK*, *HNRNPUL1*, *HNRNPF*, *HNRNPH1*, *HNRNPR*, *HNRNPAB*, and *HNRNPD*; *p* < 0.05 to *p* < 0.0001, Wilcoxon ranked sum test, Bonferroni correction). Among individual NDD *HNRNP*s, no significant differences are observed by two-way ANOVA for the contribution of each gene, highlighting their shared expression.

Hierarchical clustering shows that gene expression in the developing cortex is highly correlated among specific *HNRNP*s. Strong positive correlations are seen between *HNRNPD* and *HNRNPH3* (Pearson’s *R* = 0.87, *p* = 1.9e−15), *HNRNPU* (Pearson’s *R* = 0.86, *p* = 3e−15), *HNRNPR* (Pearson’s *R* = 0.83, *p* = 1.4e−13), and *HNRNPK* expression patterns (Pearson’s *R* = 0.8, *p* = 6.1e−12), *HNRNPR* and *HNRNPK* expression patterns (Pearson’s *R* = 0.83, *p* = 2.7e−08), *HNRNPUL1* and *HNRNPH3* expression patterns (Pearson’s *R* = 0.82, *p* = 9.5e−13), *HNRNPU* and *HNRNPH3* expression patterns (Pearson’s *R* = 0.83, *p* = 4e−13), and *HNRNPH3* and *HNRNPR* (Pearson’s *R* = 0.83, *p* = 4.1e−13) and *SYNCRIP* expression patterns (Pearson’s *R* = 0.83, *p* = 1.8e−13). While there are positive correlations between almost all NDD *HNRNP* gene expression patterns, the differences in expression along with protein homology likely play a role in the phenotypes observed.

Finally, we assessed the NDD *HNRNP* expression among specific brain regions during development (Fig. 5d). Expression is enriched in the striatum in early fetal development (*p* = 0.002, Fisher’s exact test, Benjamini-Hochberg correction) and the amygdala (*p* = 2.6e−4, Fisher’s exact test, Benjamini-Hochberg correction) during early-mid fetal development. *HNRNPK*, *HNRNPR*, *SYNCRIP*, *HNRNPF*, and *HNRNPU* are expressed in the striatum while *HNRNPH1*, *HNRNPAB*, *HNRNPUL1*, *HNRNPH3*, and *HNRNPU* are expressed in the amygdala. Expression among the striatum and amygdala is likely impacted by variation in these genes, contributing to probands’ phenotypes.

### Phenotypic assessment of probands with *HNRNP* variation

The pathogenesis of variation in the *HNRNP*s, and most genes in NDDs, has previously been discussed as independent syndromes, as opposed to a spectrum of related syndromes. While there are distinctions between the 12 *HNRNP*s and their related phenotypes, there is also considerable overlap, which is expected due to the known shared targets and functionality of hnRNPs [88]. Here, we examine the phenotypic similarities and differences among probands with variation in NDD *HNRNP*s (Fig. 7 and Additional File 2: Figs. S4 and S5, Additional File 1: Table S7).

Our findings support the five reported disorders associated with *HNRNP*s—Au-Kline syndrome (AKS, *HNRNPK*), Bain-type ID (*HNRNPH2*), *HNRNPH1*-related syndromic ID, *HNRNPR*-related syndrome, and *HNRNPU*-related disorder—and we propose seven new disorders [12–18, 21–23, 25–27, 30].

#### Phenotypic comparisons and description

Overall, the phenotypes across the *HNRNP*-related disorders are significantly highly correlated (*p* > 0.05 for all pairs of *HNRNP*s except for *HNRNPF* likely due to small sample size; Fig. 7a). These correlations are observed when considering mutation type as well (Additional File 2: Fig. S4), which is more critical for missense variation as phenotypes could be due to a variety of molecular changes. Furthermore, this correlation is in line with the degree of protein homology, with those being more similar at a protein level also being more alike at the phenotypic level (Fig. 7b). We caution that ascertainment bias likely contributes to some of this, as the majority of our cohorts have DD/ID and/or ASD, and the availability of phenotypic information varies by gene and clinical referring center.

Neurobehavioral phenotypes have the most overlap among *HNRNP* genetic disorders (Fig. 7c, d, Additional File 1: Table S7). As expected, the most common phenotype among probands in our cohort is DD/ID (88.9%, *n* = 192/216, 12/12 disorders). DD/ID varies among disorders, with diagnoses ranging from 44.4 to 100% of probands but occurs at similar rates among LGD and missense probands (Additional File 2: Fig. S5). Delayed speech and language development (57.9%, *n* = 114/197, 11/12 disorders) is common among all *HNRNP* genetic disorders except *HNRNPF*-related disorder, again with similar frequency among both mutation types. Probands with Bain-type ID (*HNRNPH2*) tend to have regression more often than other *HNRNP*-related disorders (34.8%, *n* = 8/23). Seizures are reported in 45.6% of probands (*n* = 98/215, 10/12 disorders) with variation in *HNRNP*s, primarily driven by probands with *HNRNPU*-related disorder (83.1%, *n* = 69/83), who also have a significantly higher prevalence of seizures compared to other *HNRNP*-related disorders (*p* = 0.009 compared to *HNRNPD* probands, *p* = 0.009 compared to *HNRNPH2* probands, *p* = 1.89e−10 compared to *HNRNPK* probands, *p* = 0.002 compared to *SYNCRIP* probands; Fig. 7d, Additional File 1: Table S8). Seizures overall are more common among LGD probands, but only with the inclusion of *HNRNPU* and *HNRNPR* probands (OR = 2.7, 95% CI 1.5–5.2, Fisher’s exact test).

A range of brain imaging abnormalities are observed among 37% (*n* = 70/189) of probands, with abnormalities of the corpus callosum (particularly for *HNRNPR* probands) and cerebellar vermis hypoplasia (particularly among *HNRNPH1*, *HNRNPH2*, and *HNRNPR* probands, although not significantly) being most common. Behavioral diagnoses, including ASD (35.6%, *n* = 73/205, 10/12 disorders), ADHD (8.7%, *n* = 17/195, 6/12 disorders), among others, are shared across disorders. While not significantly different, ASD is more common among probands with variation in *HNRNPD* (46.2%, *n* = 6/13), *HNRNPF* (100%, *n* = 3), *HNRNPR* (44.4%, *n* = 4/9), *SYNCRIP* (57.7%, *n* = 15/26), and *HNRNPUL2* (66.7%, *n* = 4/6), while ADHD is prevalent among *HNRNPR* probands (44.4%, *n* = 4/9) and aggression is common among *HNRNPD* probands (33.3%, *n* = 4/12). *HNRNPU*-related disorder probands with LGD variants also exhibit stereotypy (21.7%, *n* = 15/69, mostly recurrent hand flapping), as do *HNRNPR*-related disorder probands (33.3%, *n* = 3/9). Abnormal movements (22.2%, *n* = 44/198) are reported in a subset of *HNRNP* genetic disorders, including abnormal gait among probands with *SYNCRIP*-related disorder (20%, *n* = 5/25), dystonia for probands with *HNRNPD*-related disorder (25%, *n* = 3/12), and poor gross motor coordination among probands with *HNRNPUL2*-related disorder (33.3%, *n* = 2/6).

Physical abnormalities appear to be specific to certain disorders. The exception to this are abnormalities of the hands and feet (36.7%, *n* = 73/199, 9/12 disorders), including small hands and feet, abnormalities of palmar creases, clinodactyly and brachydactyly (the latter two prevalent among probands with *HNRNPR*-related syndrome, 77.8%, *n* = 7/9 and 44.4%, *n* = 4/9, respectively, clinodactyly significantly more prevalent among *HNRNPR* probands compared to *SYNCRIP* [*p* = 0.02] and *HNRNPU* probands [*p* = 0.0004]), among others with less consistency. A subset of probands have growth delay (30.5%, *n* = 61/200), particularly probands with variation in *HNRNPH1* (62.5%, *n* = 5/8), *HNRNPH2* (47.8%, *n* = 11/23), *HNRNPR* (90%, *n* = 9/10, significantly more so than *HNRNPK* probands [*p* = 0.03]), and *HNRNPUL2* (50%, *n* = 3/6). Muscular abnormalities are common (46.1%, *n* = 89/193), with hypotonia (44%) occurring more often than hypertonia (6.2%). Hypertonia is relatively specific to *HNRNPUL1* variation (22.2%, *n* = 2/9). Of eye abnormalities (41.2%, *n* = 82/199), strabismus is the most common, observed frequently in *HNRNPAB*-related disorder (66.7%, *n* = 2/3), *HNRNPH1*-related syndromic ID (62.5%, *n* = 5/8), and *HNRNPR*-related syndrome (55.6%, *n* = 5/9). Hearing impairment is observed primarily among probands with *HNRNPH3* variation (66.7%, *n* = 2/3), although with only three probands its’ unclear if this is characteristic of a majority of *HNRNPH3* variants. Skeletal morphological abnormalities (29.9%, *n* = 59/197) are primarily observed among probands with AKS (*HNRNPK*, 63%, *n* = 17/27 and *HNRNPR*-related syndrome (70%, *n* = 7/10). The most common among these probands are abnormalities of curvature of the vertebral column, including scoliosis and kyphosis (*HNRNPK*: 39.1%, *n* = 9/23; *HNRNPR*: 33.3%, *n* = 3/9). Skeletal physiological abnormalities, including joint hyper-extensibility or laxity, only occur in 27.6% (*n* = 43/190) of probands over all, but are particularly common among probands with *HNRNPD*-related disorder (20%, *n* = 2/10), *HNRNPH1*-related syndromic ID (50%, *n* = 4/8), Bain-type ID (39.1%, *n* = 9/23), AKS (27.6%, *n* = 8/29), and *SYNCRIP*-related disorder (26.1%, *n* = 6/23). Cardiac abnormalities are another known AKS phenotype, occurring in half (50%, *n* = 15/30) of probands with AKS, but only 19.4% (*n* = 38/196) of all *HNRNP* genetic disorder probands [13]. Genitourinary abnormalities (21.5%, *n* = 43/200) are a known AKS phenotype (63.3%, *n* = 19/30) and occur significantly more often among probands with AKS (*p* = 0.02 compared to Bain-type ID, *p* = 0.0001 compared to *HNRNPU*-related disorder). The most common genitourinary phenotypes observed in AKS are vesicoureteral reflux (26.7%, *n* = 8/30) and hydronephrosis (16.7%, *n* = 5/30). Enuresis is specific to *HNRNPD*-related disorder (23.1%, *n* = 3/13), while hypospadias is mostly specific to *HNRNPH1*-related syndromic ID (40% of missense variants, *n* = 2/5).

Dysmorphic facies (56.8%, *n* = 113/199) tend to not be consistent, although a few are seen among multiple disorders, including ear abnormalities (AKS, *HNRNPR*-related syndrome, *HNRNPD*-related disorder, and *HNRNPU*-related disorders), small nasal alae (*HNRNPH1*-related syndromic ID and AKS), microcephaly (AKS, *HNRNPH1*-related disorder, *HNRNPR*-related syndrome, and *HNRNPU*-related disorder), and round faces (*HNRNPD*-related disorder, Bain-type ID, and *HNRNPR*-related syndrome) (Additional File 1: Table S7). *HNRNPD*-related disorder most consistently results in a round face (23.1%, *n* = 3/13) and large ears (25%, *n* = 3/12). *HNRNPH1*-related syndromic ID probands tend of have hypoplastic alae (25%, *n* = 2/8). Bain-type ID probands tend to have abnormal palpebral fissures (22.7%, *n* = 5/22), consistent with the almond-shaped eyes previously reported for this syndrome. AKS dysmorphic features have been previously described and also consist of cleft palate (31%, *n* = 9/29), downturned corners of the mouth (20.7%, *n* = 6/29), an open bite (20.7%, *n* = 6/29), teeth abnormalities (17.2%, *n* = 5/29), a wide nasal bridge (31%, *n* = 9/29), shallow orbits/prominent eyes (20%, *n* = 6/30 each), long palpebral fissures (41.4%, *n* = 11/26), ptosis (30%, *n* = 9/30), abnormal eyebrows (20.7%, *n* = 6/29), metopic ridging (24.1%, *n* = 7/29), and abnormal nipples (20%, *n* = 6/30), all of which are more prevalent among probands with LGD variants [13]. *HNRNPR* probands have a large range of facies as well, including micro/retrognathia (50%, *n* = 4/8), teeth abnormalities (50%, *n* = 4/8), anteverted nares (37.5%, *n* = 3/8), a low hanging columella (25%, *n* = 2/8), a narrow forehead (37.5%, *n* = 3/8), up-slanted palpebral fissures (37.5%, *n* = 3/8), unusual hair (37.5%, *n* = 3/8), a broad and short neck (22.2%, *n* = 2/9), and brachycephaly (22.2%, *n* = 2/9).

#### Other *HNRNP* genes

In addition to *HNRNP*s with multiple cases of disruptive DNVs, we identified seven with less than three cases: *HNRNPA0* (*n* = 2, missense), *HNRNPA1* (*n* = 1, missense), *HNRNPA3* (*n* = 1, missense), *HNRNPA2B1* (*n* = 2, LGD), *HNRNPC* (*n* = 2, 1 LGD and 1 missense), *HNRNPLL* (*n* = 1, missense), and *PTBP2* (*n* = 1, missense) (Additional File 1: Table S10). Four of these seven (*HNRNPA0*, *HNRNPA3*, *HNRNPA1*, and *HNRNPA2B1*) are in a group closely related to an NDD hnRNP group (group 2; Fig. 1), suggesting that they may be implicated in NDDs, but mutations occur much less frequently. These were not included in the de novo or phenotypic statistical analyses but may be of importance for future screening of patients for candidate NDD-related genes.

### Comparison to similar disorders

**Fig. 8:**
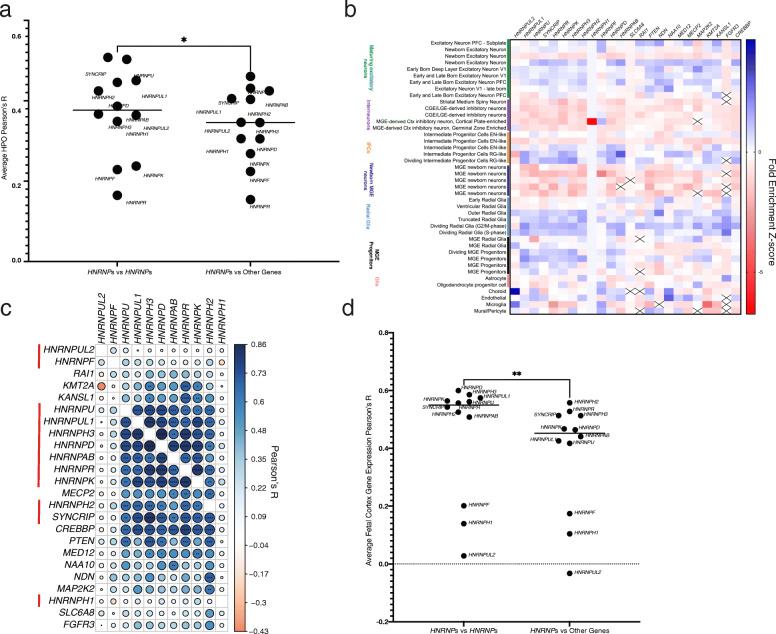
Relationship of *HNRNP*-related disorders to each other and to similarly presenting disorders. **a** Comparison of average number of HPO terms shared within *HNRNP*s versus with other similarly presenting disorders, as determined by PhenPath. The *HNRNP*-related disorders present more similarly to each other than other NDDs. HPO terms are in Table S12. **b** Heatmap showing fold change of expression of each NDD *HNRNP* and similarly presenting NDD based on HPO terms among 48 different cell types. Blue indicates increase in fold change of expression and red indicates decreased expression, as determined by *Z*-scores. **c** Correlation plot of developing fetal cortex gene expression for *HNRNP*s and genes implicated in similarly presenting disorders. Pearson correlation R values shown visually, with darker and larger circles indicating higher Pearson R values. *HNRNP*s are noted with a red line. P values were corrected by number of genes [28] and number of cell types [48]. *p < 0.05, **p < 0.01, ***p < 0.001, ****p < 0.0001. **d** Comparison of expression Pearson’s *R* values within *HNRNP*s and compared to similarly presenting disorders. The expression of the *HNRNP*s is more similar to each other than to other NDDs

It is plausible that similarities observed between *HNRNP*-related disorders may be due to ascertainment and that our *HNRNP*-related disorders may actually have more similarities to other known genetic disorders. To address this, we compared each disorder’s characteristic features to those of known NDDs (Fig. 7). HPO terms that occur in 20% or more probands were entered into the PhenPathTOOL and genes sharing the most HPO terms were compared to the expression of the *HNRNP* (Additional File 1: Table S12) [86]. HPO terms are shared with *SLC6A8* (cerebral creatine deficiency syndrome 1), *CREBBP* (Rubenstein-Taybi syndrome), *MECP2* (Rett syndrome, mental retardation, X-linked syndromic, Lubs type), *KANSL1* (Koolen-de Vries syndrome), *RAI1* (Smith-Magenis syndrome), *PTEN*, *SNRPN*, *MAP 2K2*, *FGFR3*, *KMT2A*, *MED12*, *NDN*, and *NAA10*. Notably, AKS has been suggested to be similar to Kabuki syndrome, although they do not share the most HPO terms. Additionally, methylation analyses have shown that these disorders have distinct profiles, suggesting that their shared clinical features are likely not simply due to shared genetic causes (Au et al., ASHG poster 3117). The number of genes with similar phenotypes that overlap between the *HNRNP*s highlights their shared phenotypic spectra. While there are similarities with other NDDs, correlation of HPO terms present for each disorder is higher among the *HNRNP*-related disorders, suggesting they may have shared molecular pathogenesis (*p* = 0.0013, paired *t*-test, Fig. 8a).

We assessed if shared gene expression is seen among similarly presenting disorders in developing cortical tissues (Fig. 8b, Additional File 2: Fig. S6). While there is a positive correlation (Pearson’s *R* > 0.5) among almost all genes in our analyses, correlations between genes with shared HPO terms are lower than those among the *HNRNP*s (Fig. 7c). This suggests that, while there are some shared expression patterns that are likely involved in neurodevelopment broadly, the *HNRNP*-related genetic disorders are indeed a unique family based on gene expression.

## Discussion

The goal of this study was to investigate genotype–phenotype correlations in the context of a molecularly related gene family. We selected the *HNRNP*s because they have been implicated in NDDs due to rare missense and LGD variation, as well as neurodegenerative disease and cancers primarily through changes in function, expression, and/or localization (Table 1) [8, 9, 12–14, 18, 20–23, 25, 27, 28, 89–103]. In addition, *HNRNP*s are candidates for haploinsufficiency for multiple contiguous gene deletion syndromes resulting in NDDs (*HNRNPK*, *SYNCRIP*, and *HNRNPU*, and likely *HNRNPD* and *HNRNPR*). More recently, SNVs and indels in *HNRNPU*, *HNRNPK*, *HNRNPH1/2*, and *HNRNPR* have been reported in probands with NDDs, and *HNRNPU* and *SYNCRIP* show an excess of DNVs in individuals with NDDs from recent large-scale sequencing studies [11–18, 20–23, 25–27, 35–39, 41, 58, 60, 104–106]. Thus, we hypothesized that there are (1) similar phenotypic (clinical and molecular) spectra across these disorders due to shared structure and function and (2) additional *HNRNP*-related disorders yet to be observed.

From our analyses, we identified 12 *HNRNP*s as particularly relevant to NDDs, which we term NDD *HNRNP*s: *HNRNPAB*, *HNRNPD*, *HNRNPF*, *HNRNPH1*, *HNRNPH2*, *HNRNPH3*, *HNRNPK*, *HNRNPR*, *SYNCRIP*, *HNRNPU*, *HNRNPUL1*, and *HNRNPUL2*. Multiple lines of evidence, including protein homology, expression analysis, and mutation intolerance, indicate that genes/proteins within this group are more molecularly related and more sensitive to variation than the other *HNRNP* family members. In addition to the five *HNRNP*s previously implicated in NDDs, we report seven new *HNRNP*s as candidates for emerging disorders and have integrated and compared their phenotypic features (Fig. 7, Additional File 1: Tables S7) [19, 38, 66, 104].

From over 80,000 individuals, we identify 225 probands (115 novel) with likely pathogenic SNVs and indels among the NDD *HNRNP*s—almost all of which with known inheritance are de novo (Table 2, Fig. 5). This study, thus, increases the sample size for de novo analyses by almost eightfold when compared to previous surveys, identifying a third gene family member, *HNRNPD*, that achieves nominal significance for an excess of de novo LGD or missense mutations in comparison to chimpanzee– and macaque–human mutation models (Fig. 4a). With a larger sample size, we predict that most of these NDD *HNRNP*s will ultimately become significantly associated with an excess of DNVs. Due to the limitations of these models and modes of ascertainment, many variants were not considered in de novo analyses (*n* = 147/225 not in analyses); therefore, we are underestimating the significance of variation in these genes. Overall, in large cohorts evolutionary mutational modeling provides a valuable resource for gene discovery and statistical support for NDD genes but still may miss rare disorders that are observed in the clinic. Additionally, we identified multiple disorders (*HNRNPF*-, *HNRNPH3*-, *HNRNPUL1*-, and *HNRNPUL2*-related disorders) that were not identified in the clinic or by previous statistical analyses, but by comparison to known NDDs. This highlights the need for intersection between statistical modeling, clinical identification, and novel approaches to identifying NDD candidate genes to uncover the rarest of disorders.

Our results support emerging syndromes associated with seven genes and expand upon known *HNRNP*-related disorders [10, 19, 38, 66, 104]. We are able to explain some of the phenotypic similarities and differences based on protein homology, gene expression—which is highly correlated among almost all *HNRNP*s—and known shared function. At cell type resolution, NDD *HNRNP* expression is most strongly enriched among progenitor populations of the human cerebral cortex and MGE, including mitotic radial glia, and their expression is depleted in both early-born excitatory neurons and newborn MGE neurons (Fig. 6b, Additional File 2: S3A,B). Radial glia, one of the earliest cells found in brain development, act as neural stem cells of the cerebral cortex and are important in the differentiation and migration of many neuronal cell types. Enriched expression of NDD *HNRNP*s in actively dividing radial glia suggests a possibility that disruption of hnRNPs may lead to aberrant neuronal development. Disruption of radial glia can happen at many steps in early development, including changes in proliferation leading to differing number of neurons, changes in scaffolding leading to impaired neuronal migration, and subtler defects in neurite extension, synaptogenesis, and neuronal connectivity [107–109]. These types of changes have been detected in ASD, DD/ID, epilepsy, and schizophrenia. Interestingly, some NDD hnRNPs have already been implicated in cell division through cancer studies, including hnRNPD, hnRNPK, SYNCRIP, and hnRNPU [110–114]. Thus, it is plausible that altered cell division, differentiation, and neuronal migration may be playing a role in the pathogenesis of *HNRNP* variation. At a brain region level, we see enrichment in the early fetal striatum and the early-mid fetal amygdala, both of which have been implicated in NDDs [1, 115–117]. At the adult tissue level, it is clear that *HNRNPK* has the highest and most ubiquitous expression, which may account for some of the more physical phenotypes observed in probands with AKS. hnRNPR is also known to regulate *HOX* genes, which likely plays a role in the physical manifestations seen in *HNRNPR*-related disorder, and dysregulation of *HOX* genes may be important to other *HNRNP*-related disorders as well [18]. Overall, we observe that missense variants cluster for all hnRNPs more so among probands than controls, and especially for hnRNPH2, hnRNPK, hnRNPR, and hnRNPUL1, and that particular protein domains appear to be impacted, including RNA- and DNA-binding domains.

Consistent with our ascertainment, DD/ID are the most prevalently shared features among all probands with *HNRNP*-related disorders, which may be due to ascertainment bias (Fig. 7). Over half have speech delay and over 40% have seizures and/or hypotonia, while over one third each have structural brain abnormalities or ASD. Structural brain abnormalities include abnormalities of the corpus callosum, cerebellar vermis hypoplasia, and dysmorphic ventricles, which may be explained by the high radial glia expression of *HNRNP*s. Most have some dysmorphic features, and several have a range of physical abnormalities impacting the cardiac, skeletal, and genitourinary systems, although generally these are inconsistent, except for AKS and *HNRNPR*-related syndrome, which have been previously described (Additional File 1: Table S7) [13, 18].

While there is subjectively a pattern of phenotypes, we also observe a strong correlation between almost all of the *HNRNP*-related disorders, as hypothesized given they have similar molecular structure and function and shared gene expression (Fig. 7a). Notably, we observe that phenotypic correlations follow a similar pattern to those of protein homology, suggesting that more similar proteins molecularly do have more similar consequences when disrupted (Fig. 7b). *HNRNPF*-related disorder has the weakest correlations, although this is likely due to (1) lower sample size (*n* = 3) with limited phenotypic information and (2) a potential association with autism without intellectual disability (*n* = 1/2 with ASD and no DD/ID) [34]. There are similar patterns in correlations based on LGD and severe missense variation, highlighting the shared impacts of the two types of variants on each gene (Additional File 2: Fig. S4). While we have far fewer probands with missense variation, they typically have a less severe phenotype, and their pathogenic significance remains to be determined given the small sample size.

Overall, our phenotypic analyses of the previously reported *HNRNPU*-related disorder, AKS, Bain-type ID, *HNRNPH1*-related syndromic ID, and *HNRNPR*-related syndrome are consistent with what has been published [12, 13, 18, 20–23, 25–27, 60]. We generally find that the phenotypic spectrum of newly recruited patients matches what has previously been reported for LGD mutations, although the phenotypic consequences of missense variation are less well established. Only a few differences were observed. One is that our data do not support the previous suggestion that probands with *HNRNPU*-related disorder have renal and cardiac abnormalities, or these phenotypes have much lower penetrance. Another is that ASD has a higher prevalence than previously reported for AKS, particularly for missense variants. We also provide evidence phenotypically and based on gene expression that *HNRNPH1*-related syndromic ID and Bain-type ID (*HNRNPH2*) are indeed independent disorders, which has been under debate. Also supporting this are the variant types, which cluster in the M9 nuclear targeting region of hnRNPH2, likely impacting its localization and global translation based on in vitro work, but are seen across the entirety of hnRNPH1 [118, 119].

We report seven novel disorders: *SYNCRIP*-, *HNRNPD*-, *HNRNPUL1*-, *HNRNPUL2*-, *HNRNPAB*-, *HNRNPF*-, and *HNRNPAB*-related disorders. Both *SYNCRIP* and *HNRNPD* have been NDD candidates due to being in the critical region of genomic disorders (Table 3). *SYNCRIP*, having high protein homology to hnRNPR, has also been observed to have recurrent de novo LGD variants in large NDD sequencing studies [39, 69, 74]. Indeed, we see support of this both phenotypically and statistically, with *SYNCRIP* LGD variants reaching exome-wide significance by the CH model (Fig. 5). We propose that *SYNCRIP*-related disorder primarily manifests as DD/ID, ASD, ADHD, hypotonia, speech delay, and structural brain abnormalities (including abnormal/dysmorphic ventricles, abnormalities of the corpus callosum, and Chiari malformations), with some probands having abnormal gait or ataxia, hyper-extensible joints, and hand and feet abnormalities (Additional File 1: Table S7). Many SYNCRIP RNA and protein interactors are critical in neuronal RNA and membrane trafficking, synaptic plasticity, axonogenesis, neuronal morphology, and dendritic translation [120–125].

Probands with *HNRNPD*-related syndrome show a high prevalence of DD/ID, speech delay, and ASD and/or other behavioral phenotypes (Additional File 1: Table S7) [55]. These probands also have consistent facial features of a round face, epicanthus, and large ears. Other emotional disturbances are also noted, including aggression, intermittent explosive disorder, and depression. It has been suggested that the gene may play a role in the development of enkephalinergic neurons, known to be important in neurodevelopment, particularly for emotional responses and ASD, which aligns with more emotional disturbances in *HNRND* probands [126, 127]. *HNRNPD* expression is also altered following NMDA receptor stimulation, which may impact the wide range of intracellular cascades, including those involved in cell survival, differentiation, and neuroplasticity.

hnRNPUL1 and hnRNPUL2 have high protein homology to hnRNPU, although they appear to have milder phenotypes when disrupted. *HNRNPUL1*-related disorder consists of DD/ID, although about half as often as with *HNRNPU* variation, short stature, motor and speech delay, structural brain, skeletal, and cardiac abnormalities, abnormal gait, ASD, failure to thrive, and hypertonia (Additional File 1: Table S7). Previously, it was suggested that missense variation in *HNRNPUL1* contributes to “high-functioning” autism, meaning an autism diagnosis and IQ in the normal range, which our data support [34]. Missense variants cluster significantly among probands compared to controls, although not in a clear protein domain, suggesting that disruption within that part of the protein may be responsible for some phenotypes (Fig. 4). Probands with *HNRNPUL2*-related disorder exhibit DD/ID, ASD, and motor and speech delay. *HNRNPUL2* gene expression has also been shown to be significantly decreased in the blood of first episode psychosis patients, suggesting a neuronal functional role and that we may have under-ascertained individuals with neuropsychiatric phenotypes [128]. Although understudied, both *HNRNPUL1* and *HNRNPUL2* are known to be critical in the DNA damage response, which has been associated with neurodevelopment [129–132].

Three of our novel disorders only have three probands each: *HNRNPAB*-, *HNRNPF*-, and *HNRNPH3*-related disorders, making it difficult to draw conclusions. For probands with *HNRNPAB* variants, DD/ID is common, as are delayed speech and language, ASD, hypotonia, and strabismus. Little is known about hnRNPAB outside of cancer, but it has been shown to regulate expression of neurodevelopmental genes and those involved in glutamate signaling, as well as neural cell motility [133, 134]. Probands with *HNRNPH3*-related disorder have a high prevalence of DD/ID, like the other *HNRNPH* disorders, although they manifest characteristic hearing loss as well. However, the gene expression is shared with both other *HNRNPH* genes, suggesting that their shared phenotypes may have similar molecular causes and that additional probands may reveal a stronger phenotypic relationship. hnRNPF is highly homologous to hnRNPH1/2/3 (Fig. 2, group 7), and like those genes, we identified only reports of severe missense variation. Thus, *HNRNPF*-related disorder may have more molecular similarities to *HNRNPH*-related disorders, although with only three probands this is difficult to conclude. All probands have ASD, and half have DD/ID, suggesting that autism with a normal IQ may be an associated phenotype as previously suggested (Table S7) [34]. While little is known about the specific function of hnRNPF, it is has been shown to interact with other hnRNPH proteins and regulate neuronal-specific splicing, as well as myelin basic protein synthesis in oligodendrocytes [135, 136]. The lack of LGD variants in our cohort as well as gnomAD for *HNRNPH2*, *HNRNPH3*, and *HNRNPF* may suggest that loss-of-function mutations are embryonic lethal.

As there are some shared features among NDDs overall, we wanted to ensure that the common phenotypes and expression were specific among our *HNRNP*-related disorders compared to similarly presenting disorders. While several disorders share multiple HPO terms with our *HNRNP*-related disorders, the *HNRNP*-related disorders are more similar to each other than to other NDDs. It is plausible that similarly presenting disorders share disrupted gene regulation with the *HNRNP*-related disorders, explaining some shared phenotypes. Based on gene expression in the developing fetal cortex, the expression of the *HNRNP*s are more similar to each other than similarly presenting disorders (Fig. 8c,d). Thus, we conclude that, while of course there are many disorders that may share phenotypes, the *HNRNP*-related disorders are unique in their presentation and gene expression, as well as protein function, supporting a gene family approach.

Our study highlights the value and challenge of integrating data across multiple centers and the published literature for a group of highly heterogeneous NDDs. A strength is our large sample size of > 80,000 individuals, pulling from published work, our own unpublished resources, and international collaborations, but for rare disorders this is still insufficient. As shown by our de novo mutation analyses, the sample sizes used are not high enough to detect evolutionary evidence that variation in many of these genes is pathogenic, but based on clinical patterns, protein homology, and gene expression, there is reason to conclude pathogenicity. Multiple *HNRNP*s have been implicated in autism with normal IQ and/or neuropsychiatric disorders that have little effect on IQ, which are severely under-ascertained not only in this study but across the board in NDD research. Additionally, the average age of patients in our study is 10.5 years, suggesting that any phenotypes associated with adults are likely missed. Thus, while much of our data shows that there are not many significant differences among *HNRNP*-related disorders, this is likely not the full story of these disorders. Much work is needed to establish the molecular phenotypes to fully understand the pathophysiology of these disorders, which will determine if there are shared pathways affected by these disorders and inform potential treatment strategies.

## Conclusions

In summary, the hnRNPs are a family of proteins that have been shown repeatedly to impact neurological disease. Here, we establish both a statistical enrichment of DNVs and phenotypic evidence that many of the *HNRNP*s are involved in NDDs. The conservation, shared expression, and phenotypic consequences clearly support a subset of these as high likelihood candidates of NDD when mutated. More work is necessary to understand the molecular underpinnings of NDD-related *HNRNP* variation, especially since compounds utilized in diseases such as addiction and cancer are available to target, both up- and downregulating, a subset of hnRNPs [83, 137]. Future work may identify hnRNP-targeting compounds and/or downstream-targeting compounds that could prove to be efficacious in modifying patient outcomes. With the shared function, localization, and binding targets of many of the NDD hnRNPs, it is likely that one compound may benefit probands with changes in related genes. Using the hnRNPs as an example, we have provided an evidence-based foundation supporting that, by identifying relevant NDD gene families and distinguishing molecularly related subgroups, characterization of de novo mutated genes among NDDs can be performed more effectively. This facilitates improved diagnosis and prospective assessment of NDDs as well as potential future development of more impactful therapeutics, while also providing information on neurodevelopment as a whole.

## Supplementary Information

**Additional file 1: Supplementary Tables S1-S12. Table S1.** Cohorts utilized in current study. **Table S2.** Pathogenicity predictions by gnomAD and GeVIR for each hnRNP. **Table S3.** Significant *p* values of two-way ANOVA between cell type and transcripts per million (TPM) for each NDD *HNRNP’*s GTEx data. **Table S4.** *P* values and fold change for scRNA data from developing human cortex. **Table S5.** Significant p values of two-way ANOVA between cell type and fold expression for each *HNRNP*. **Table S6.** De novo enrichment and CLUMP analyses of current cohort. **Table S7.** Phenotypes among *HNRNP*-related disorders. **Table S8.** Uncorrected p values for pairwise Fisher’s exact tests for each phenotype occurring in 20% of an *HNRNP* group. **Table S9.** Uncorrected p values for phenotype correlations between each *HNRNP*-related disorder. **Table S10.** Variants and phenotypes for *HNRNP*s with < 3 probands. **Table S11.** Probands with copy number variants. **Table S12.** Shared HPO terms with similarly presenting genetic disorders.

**Additional file 2: Supplementary Figures S1-S6. Fig. S1.** Dendrogram of hnRNPs based on multiple sequence alignment of canonical amino acid sequences. Colors match those seen in Fig. 2. NDD hnRNPs are shown in black boxes. **Fig. S2.** Pathogenicity assessment of variation in hnRNPs. A) Gene Variation Intolerance Ranking (GeVIR), loss-of-function observed/expected upper bound fraction (LOEUF), and Variation Intolerant Region Loss-of-Function (VIRLoF) percentiles. Average LOEUF percentile is significantly higher for NDD *HNRNP*s (*n* = 13) compared to other *HNRNP*s (*n* = 15). B) Average fold change for GeVIR, LEOUF, and VIRLoF for autosomal dominant (AD) and autosomal recessive (AR) variants. Average LEOUF fold change for AD mutations is significantly higher for NDD *HNRNP*s compared to other *HNRNP*s, with the AD VIRLoF fold change trending in the same direction. The AR LEOUF fold change is trending towards being significantly higher among other *HNRNP*s compared to NDD *HNRNP*s. One-way t-test. * *p* < 0.05. **Fig. S3.** Expression of *HNRNP*s among adult tissues and the developing human cortex. A) Heatmap of all *HNRNP* expression in developing cortex tissues. B) Comparison of fold expression of NDD *HNRNP*s to non-NDD *HNRNP*s. C) Heatmap of all *HNRNP* expression (transcript level expression) in adult brain tissues from GTEx. D) Heatmap of NDD *HNRNP* expression (transcript level expression) in all tissues from GTEx. **Fig. S4.** Phenotypic correlations for LGD and missense variant probands. A) Correlation matrix of phenotypes across *HNRNP* probands with LGD variation (genes with only missense variation excluded) and B) severe missense variation (genes with only LGD variation excluded. * p < 0.05; ** *p* < 0.01; *** *p* < 0.001; **** *p* < 0.0001. **Fig. S5.** Phenotypic comparisons between LGD and missense variants by *HNRNP*. **Fig. S6.** GTEx expression of NDD *HNRNP*s and genes associated with similarly presenting disorders.

**Additional file 3:** **Table S13.** GenBank accession numbers.

## Data Availability

All data from probands generated or analyzed during this study are included in this published article and its supplementary information files. Accession numbers can be found in Additional File 3: Table S13 [138]. All other data is available from previously published resources. **Web Resources** ape: http://ape-package.ird.fr/ CADD: https://cadd.gs.washington.edu/ ClinVar: https://www.ncbi.nlm.nih.gov/clinvar/ Clustal Omega: http://clustal.org/omega corrplot: https://github.com/taiyun/corrplot corrr: https://github.com/tidymodels/corrr CLUMP: https://github.com/tycheleturner/clump CSEA: http://genetics.wustl.edu/jdlab/csea-tool-2/ DECIPHER: http://depher.sanger.ac.uk/ denovo-db: http://denovo-db.gs.washington.edu Ensembl VEP (hg38): http://uswest.ensembl.org/Homo_sapiens/Tools/VEP?db=core GeneMatcher: http://genematcher.org GenBank: https://www.ncbi.nlm.nih.gov/genbank/ GeVIR: http://www.gevirank.org/ gnomAD: https://gnomad.broadinstitute.org/ GTEx: https://gtexportal.org/ MyGene2: https://mygene2.org/MyGene2/ MetaDome: https://stuart.radboudumc.nl/metadome/ NMDEscPredictor: https://nmdprediction.shinyapps.io/nmdescpredictor/ PhenPath: http://phenpath.biocomp.unibo.it/phenpath/ RVAideMemoire: https://CRAN.R-project.org/package=RVAideMemoire SFARI Base: https://base.sfari.org SPARK: https://sparkforautism.org/ UCSC Cell Browser: https://cells.ucsc.edu Uniprot: http://uniprot.org

## Abbreviations

ADHD: Attention deficit hyperactivity disorder
AKS: Au-Kline syndrome
ALS: Amyotrophic lateral sclerosis
ANOVA: Analysis of variance
ASD: Autism spectrum disorder
BGL: Baylor Genetics Laboratory
CH model: Chimpanzee–human divergence model
CLUMP: CLUstering by Mutation Position
CNV: Copy number variant
DD: Developmental delay
DDD: Deciphering Developmental Disorders
DNA: Deoxyribonucleic acid
DNV: De novo variant
FTLD: Frontotemporal lobar degeneration
GERD: Gastroesophageal reflux disease
GeVIR: Gene Variation Intolerance Rank
hnRNP/*HNRNP*: Heterogeneous nuclear ribonucleoprotein (hnRNP for protein, *HNRNP* for gene)
HPO: Human Phenotype Ontology
ID: Intellectual disability
LGD: Likely gene disrupting
MIS: Missense
MIS20: Missense variant with CADD score ≥ 20
MIS30: Missense variant with CADD score ≥ 30
NDD: Neurodevelopmental disorder
NMDA: *N*-methyl-d-aspartate receptor
pLI: Probability of loss of function
RNA: Ribonucleic acid
SEA: Specific Expression Analyses
SNV: Single-nucleotide variant
