# Supplementary material for: Rare deleterious mutations of HNRNP genes result in shared neurodevelopmental disorders
Source: Genome Med. 2021 Apr 19;13:63. doi: 10.1186/s13073-021-00870-6 (PMC8056596; doi:10.1186/s13073-021-00870-6)
Supplement: Supplementary file 2 — Additional file 2: Supplementary Figures S1-S6. Fig. S1. Dendrogram of hnRNPs based on multiple sequence alignment of canonical amino acid sequences. Colors match those seen in Fig. 2. NDD hnRNPs are shown in black boxes. Fig. S2. Pathogenicity assessment of variation in hnRNPs. A) Gene Variation Intolerance Ranking (GeVIR), loss-of-function observed/expected upper bound fraction (LOEUF), and Variation Intolerant Region Loss-of-Function (VIRLoF) percentiles. Average LOEUF percentile is significantly higher for NDD HNRNPs (n = 13) compared to other HNRNPs (n = 15). B) Average fold change for GeVIR, LEOUF, and VIRLoF for autosomal dominant (AD) and autosomal recessive (AR) variants. Average LEOUF fold change for AD mutations is significantly higher for NDD HNRNPs compared to other HNRNPs, with the AD VIRLoF fold change trending in the same direction. The AR LEOUF fold change is trending towards being significantly higher among other HNRNPs compared to NDD HNRNPs. One-way t-test. * p < 0.05. Fig. S3. Expression of HNRNPs among adult tissues and the developing human cortex. A) Heatmap of all HNRNP expression in developing cortex tissues. B) Comparison of fold expression of NDD HNRNPs to non-NDD HNRNPs. C) Heatmap of all HNRNP expression (transcript level expression) in adult brain tissues from GTEx. D) Heatmap of NDD HNRNP expression (transcript level expression) in all tissues from GTEx. Fig. S4. Phenotypic correlations for LGD and missense variant probands. A) Correlation matrix of phenotypes across HNRNP probands with LGD variation (genes with only missense variation excluded) and B) severe missense variation (genes with only LGD variation excluded. * p < 0.05; ** p < 0.01; *** p < 0.001; **** p < 0.0001. Fig. S5. Phenotypic comparisons between LGD and missense variants by HNRNP. Fig. S6. GTEx expression of NDD HNRNPs and genes associated with similarly presenting disorders. [file 13073_2021_870_MOESM2_ESM.pdf]

Fig. S1

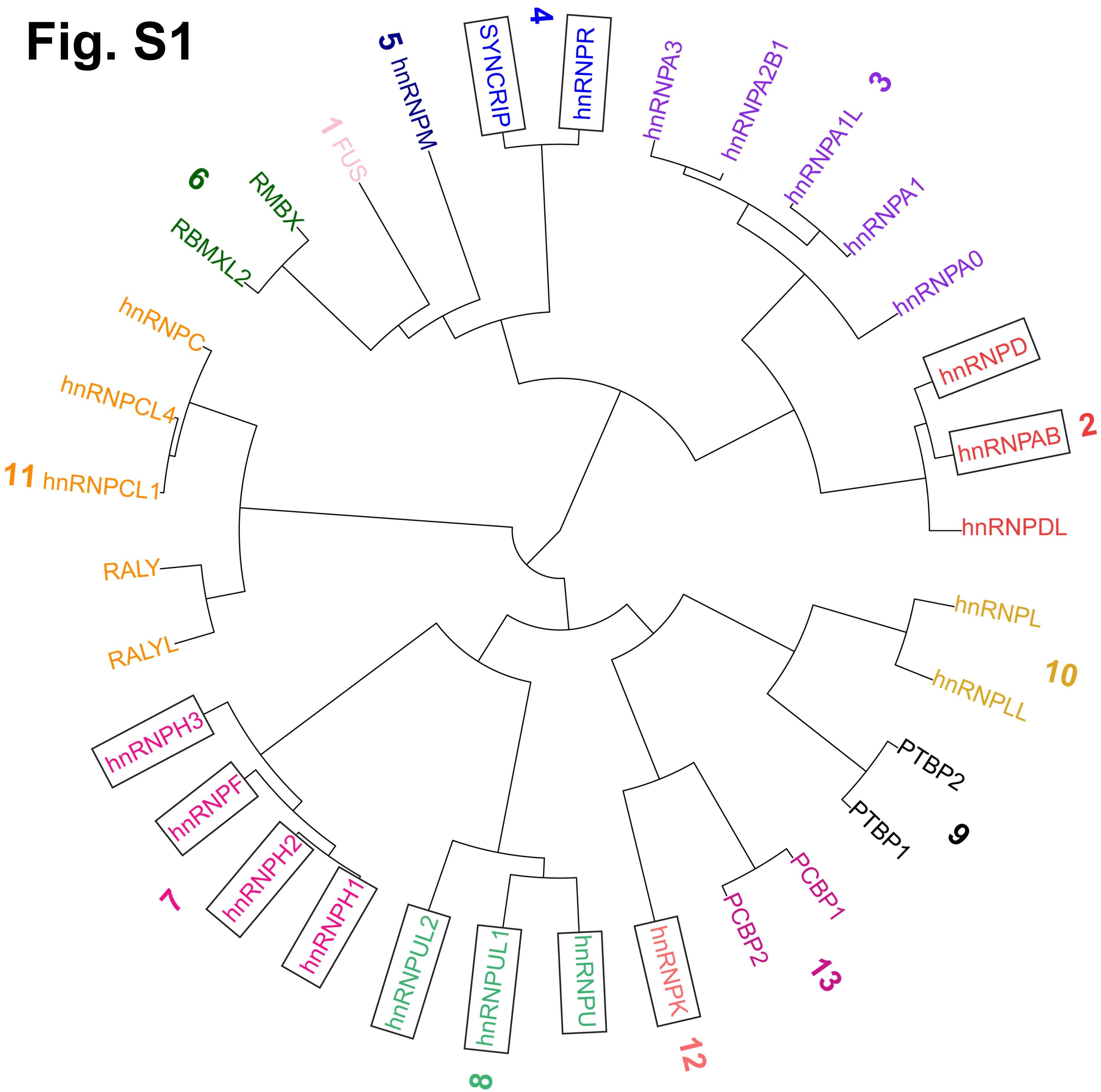

1 FUS
2 hnRNPD hnRNPAB hnRNPDL
3 hnRNPA3 hnRNPA2B1 hnRNPA1L hnRNPA1 hnRNPA0
4 SYNCRIP hnRNPR
5 hnRNPM
6 RMBX RBMXL2
7 hnRNPH3 hnRNPF hnRNPH2 hnRNPH1
8 hnRNPUL2 hnRNPUL1 hnRNPU
9 PTBP2 PTBP1
10 hnRNPL hnRNPLL
11 hnRNPC hnRNPCL4 hnRNPCL1 RALY RALYL
12 hnRNPK
13 PCBP1 PCBP2

Fig. S2

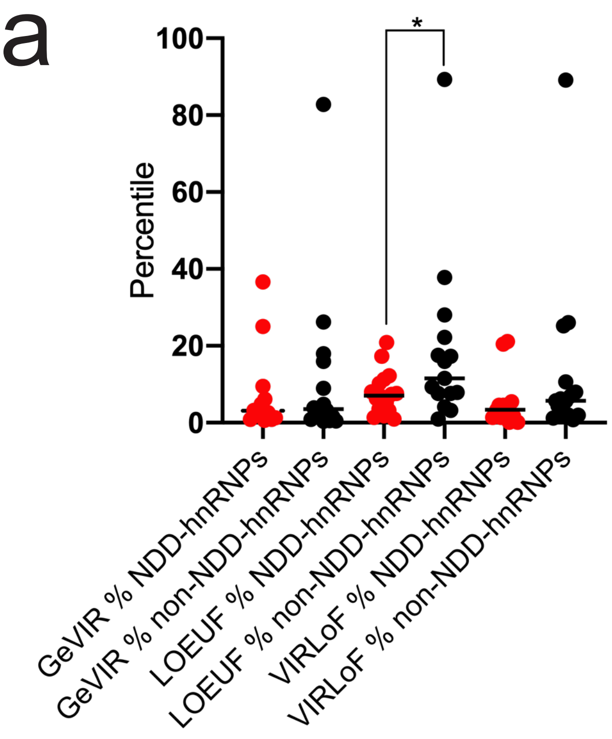

a
*
100
80
60
40
20
0
Percentile
GeVIR % NDD-hnRNPs
GeVIR % non-NDD-hnRNPs
LOEUF % NDD-hnRNPs
LOEUF % non-NDD-hnRNPs
VIRLoF % NDD-hnRNPs
VIRLoF % non-NDD-hnRNPs

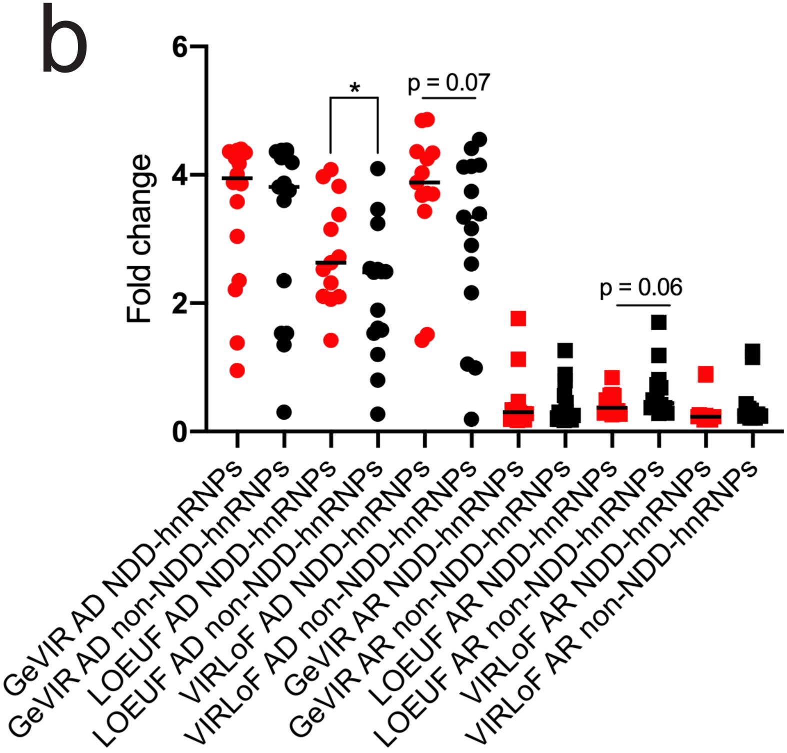

b
*
p = 0.07
p = 0.06
6
4
2
0
Fold change
GeVIR AD NDD-hnRNPs
GeVIR AD non-NDD-hnRNPs
LOEUF AD NDD-hnRNPs
LOEUF AD non-NDD-hnRNPs
VIRLoF AD NDD-hnRNPs
VIRLoF AD non-NDD-hnRNPs
GeVIR AR NDD-hnRNPs
GeVIR AR non-NDD-hnRNPs
LOEUF AR NDD-hnRNPs
LOEUF AR non-NDD-hnRNPs
VIRLoF AR NDD-hnRNPs
VIRLoF AR non-NDD-hnRNPs

Fig. S3

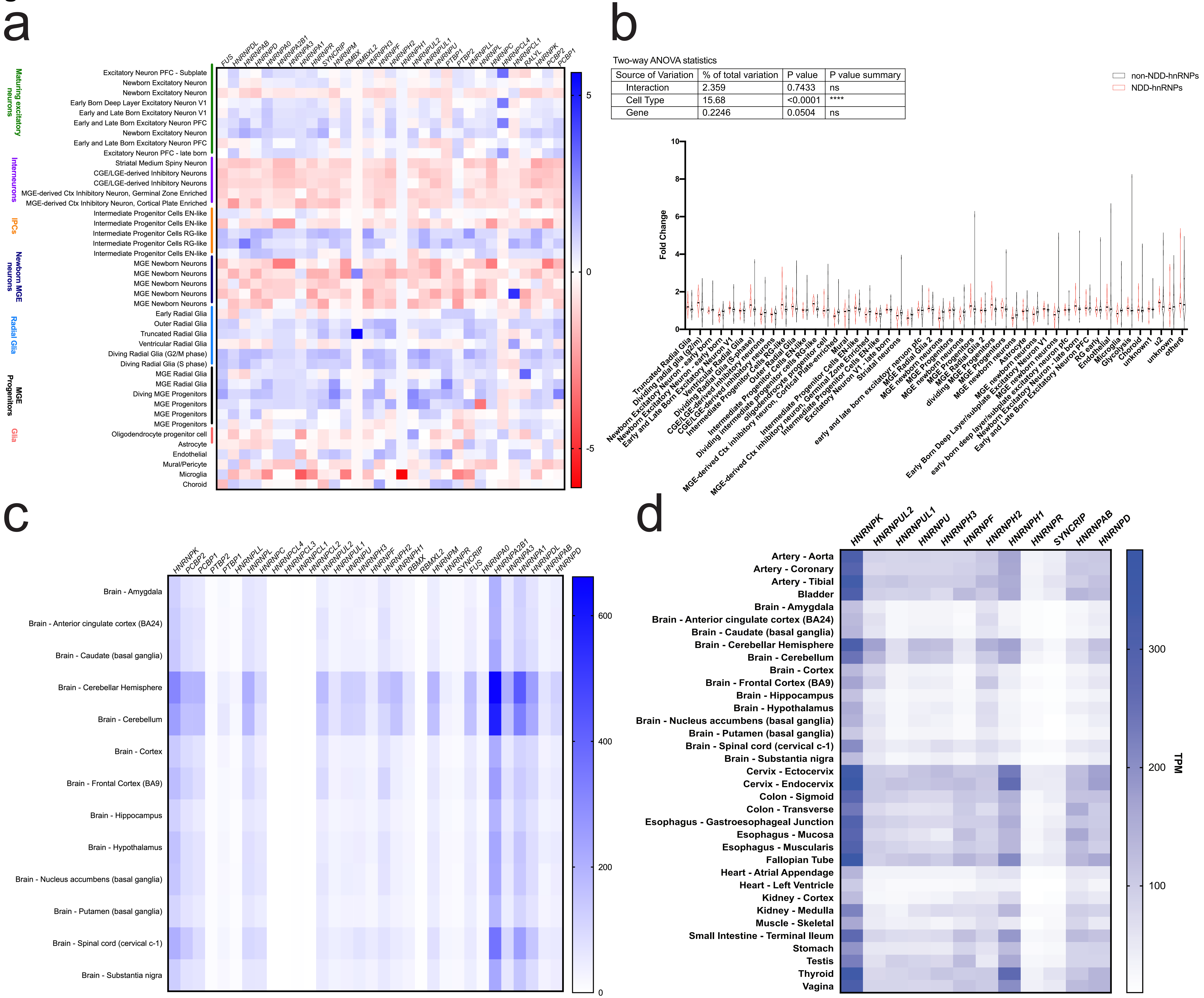

Two-way ANOVA statistics

Source of Variation	% of total variation	P value	P value summary
Interaction	2.359	0.7433	ns
Cell Type	15.68	<0.0001	****
Gene	0.2246	0.0504	ns

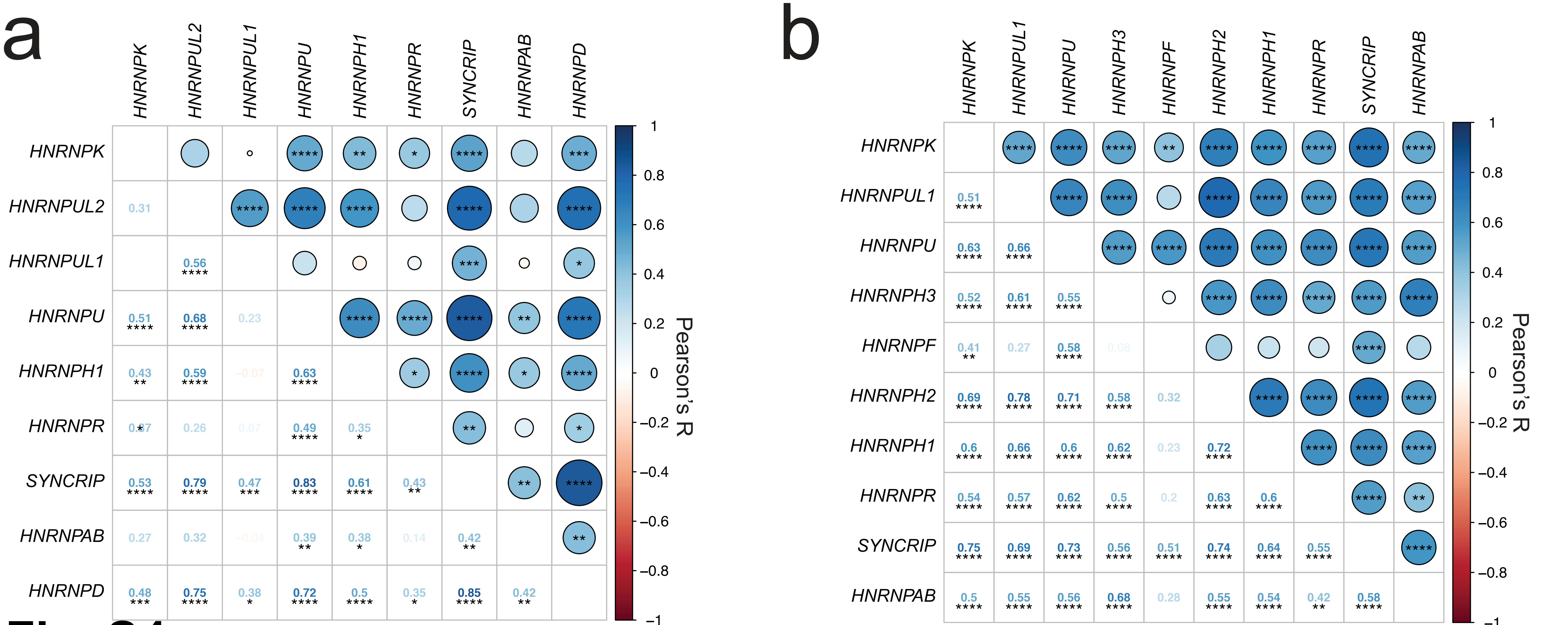

a
b
Pearson's R
HNRNPK
HNRNPUL2
HNRNPUL1
HNRNPU
HNRNPH1
HNRNPR
SYNCRIP
HNRNPAB
HNRNPD
HNRNPH3
HNRNPF
HNRNPH2

Fig. S4

Fig. S5

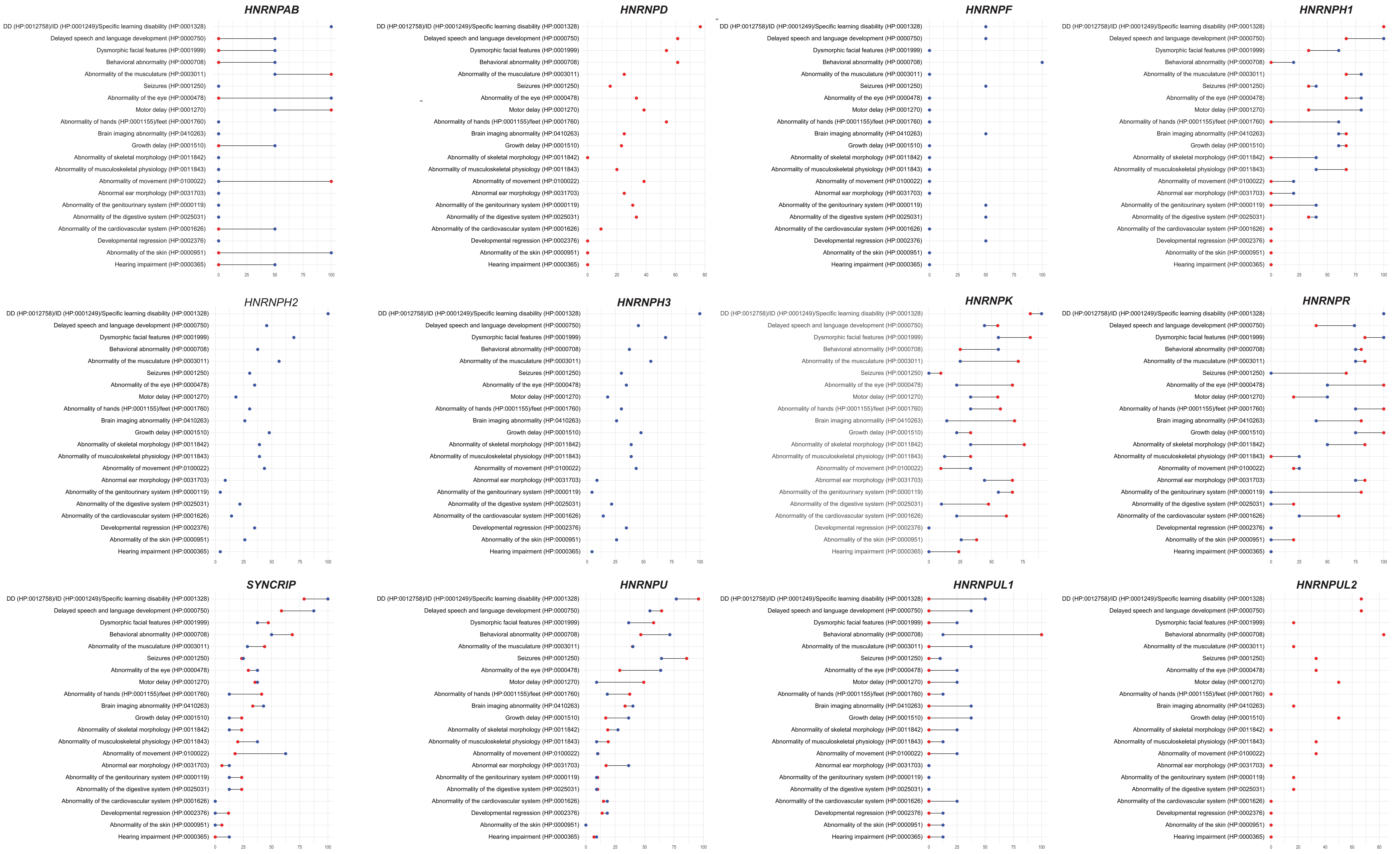
HNRNPAB
HNRNPD
HNRNPF
HNRNPH1
HNRNPH2
HNRNPH3
HNRNPK
HNRNPR
SYNCRIP
HNRNPU
HNRNPUL1
HNRNPUL2
DD (HP:0012758)/ID (HP:0001249)/Specific learning disability (HP:0001328)
Delayed speech and language development (HP:0000750)
Dysmorphic facial features (HP:0001999)
Behavioral abnormality (HP:0000708)
Abnormality of the musculature (HP:0003011)
Seizures (HP:0001250)
Abnormality of the eye (HP:0000478)
Motor delay (HP:0001270)
Abnormality of hands (HP:0001155)/feet (HP:0001760)
Brain imaging abnormality (HP:0410263)
Growth delay (HP:0001510)
Abnormality of skeletal morphology (HP:0011842)
Abnormality of musculoskeletal physiology (HP:0011843)
Abnormality of movement (HP:0100022)
Abnormal ear morphology (HP:0031703)
Abnormality of the genitourinary system (HP:0000119)
Abnormality of the digestive system (HP:0025031)
Abnormality of the cardiovascular system (HP:0001626)
Developmental regression (HP:0002376)
Abnormality of the skin (HP:0000951)
Hearing impairment (HP:0000365)

Fig. S6

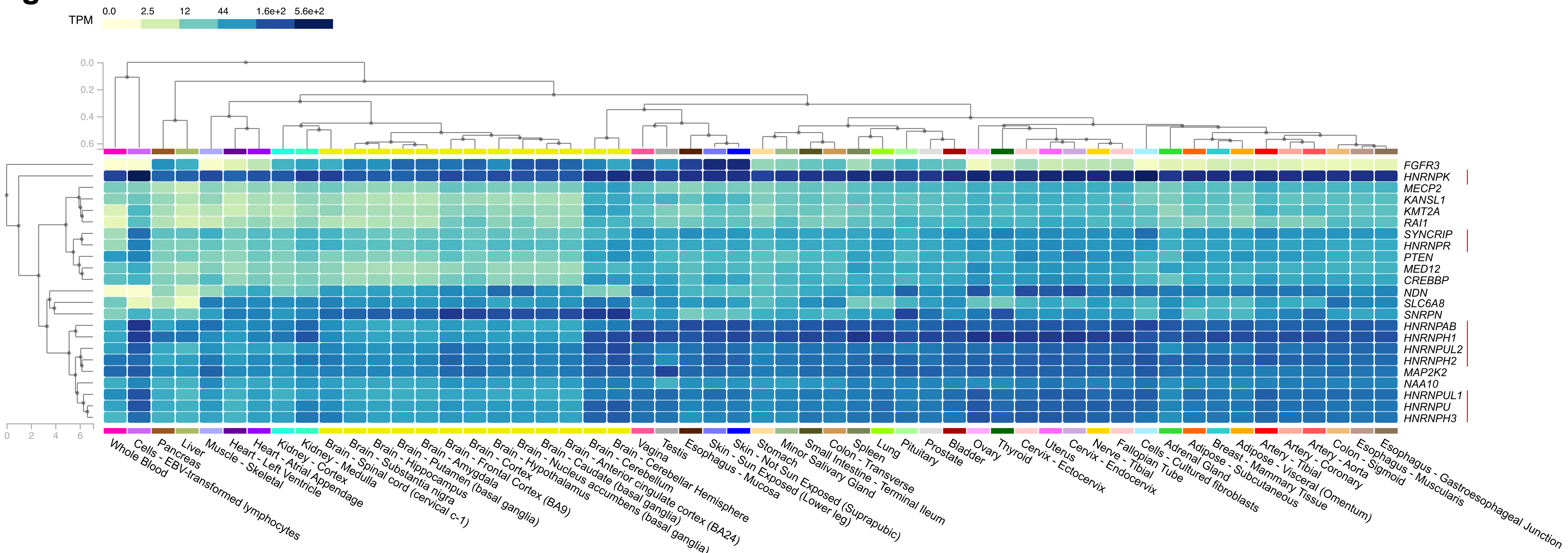
TPM
0.0
2.5
12
44
1.6e+2
5.6e+2
FGFR3
HNRNPK
MECP2
KANSL1
KMT2A
RAI1
SYNCRIP
HNRNPR
PTEN
MED12
CREBBP
NDN
SLC6A8
SNRPN
HNRNPAB
HNRNPH1
HNRNPUL2
HNRNPH2
MAP2K2
NAA10
HNRNPUL1
HNRNPU
HNRNPH3
Whole Blood
Cells - EBV-transformed lymphocytes
Pancreas
Liver
Muscle - Skeletal
Heart - Left Ventricle
Heart - Atrial Appendage
Kidney - Cortex
Kidney - Medulla
Brain - Spinal cord (cervical c-1)
Brain - Substantia nigra
Brain - Hippocampus
Brain - Putamen (basal ganglia)
Brain - Amygdala
Brain - Frontal Cortex (BA9)
Brain - Cortex
Brain - Hypothalamus
Brain - Nucleus accumbens (basal ganglia)
Brain - Caudate (basal ganglia)
Brain - Anterior cingulate cortex (BA24)
Brain - Cerebellum
Brain - Cerebellar Hemisphere
Vagina
Testis
Esophagus - Mucosa
Skin - Sun Exposed (Lower leg)
Skin - Not Sun Exposed (Suprapubic)
Stomach
Minor Salivary Gland
Small Intestine - Terminal Ileum
Colon - Transverse
Spleen
Lung
Pituitary
Prostate
Bladder
Ovary
Thyroid
Cervix - Ectocervix
Uterus
Cervix - Endocervix
Nerve - Tibial
Fallopian Tube
Cells - Cultured fibroblasts
Adrenal Gland
Adipose - Subcutaneous
Breast - Mammary Tissue
Adipose - Visceral (Omentum)
Artery - Tibial
Artery - Coronary
Artery - Aorta
Colon - Sigmoid
Esophagus - Muscularis
Esophagus - Gastroesophageal Junction

Fig. S1. Dendrogram of hnRNPs based on multiple sequence alignment of canonical amino acid sequences. Colors match those seen in Figure 2. NDD hnRNPs are shown in black boxes.

Fig. S2. Pathogenicity assessment of variation in hnRNPs. A) Gene Variation Intolerance Ranking (GeVIR), loss-of-function observed/expected upper bound fraction (LOEUF), and Variation Intolerant Region Loss-of-Function (VIRLoF) percentiles. Average LOEUF percentile is significantly higher for NDD *HNRNP*s (n = 13) compared to other *HNRNP*s (n = 15). B) Average fold change for GeVIR, LEOUF, and VIRLoF for autosomal dominant (AD) and autosomal recessive (AR) variants. Average LEOUF fold change for AD mutations is significantly higher for NDD *HNRNP*s compared to other *HNRNP*s, with the AD VIRLoF fold change trending in the same direction. The AR LEOUF fold change is trending towards being significantly higher among other *HNRNP*s compared to NDD *HNRNP*s. One-way t-test. * $p < 0.05$

Fig. S3. Expression of *HNRNP*s among adult tissues and the developing human cortex. A) Heatmap of all *HNRNP* expression in developing cortex tissues.
B) Comparison of fold expression of NDD *HNRNP*s to non-NDD *HNRNP*s. C) Heatmap of all *HNRNP* expression (transcript level expression) in adult brain tissues from GTEx. D) Heatmap of NDD *HNRNP* expression (transcript level expression) in all tissues from GTEx.

Fig. S4. Phenotypic correlations for LGD and missense variant probands.

A) Correlation matrix of phenotypes across *HNRNP* probands with LGD variation (genes with only missense variation excluded) and B) severe missense variation (genes with only LGD variation excluded. * $p < 0.05$; ** $p < 0.01$; *** $p < 0.001$; **** $p < 0.0001$.

Fig. S5. Phenotypic comparisons between LGD and missense variants by *HNRNP*.

Fig. S6. GTEx expression of NDD *HNRNP*s and genes associated with similarly presenting disorders.
