# Supplementary material for: Rare deleterious mutations of HNRNP genes result in shared neurodevelopmental disorders
Source: Genome Med. 2021 Apr 19;13:63. doi: 10.1186/s13073-021-00870-6 (PMC8056596; doi:10.1186/s13073-021-00870-6)
Supplement: Supplementary file 3 — Additional file 3: Table S13. GenBank accession numbers. [file 13073_2021_870_MOESM3_ESM.docx]

**Table S13.** GenBank accession numbers.

*HNRNPA0*: NM_006805.3; hnRNPA0: NP_006796.1

*HNRNPA1*: NM_002136.3; hnRNPA1: NP_002127.1﻿

*HNRNPA2B1*: NM_031243.2; hnRNPA2B1: NP_112533.1

*HNRNPAB*: NM_031266.2; hnRNPAB: NP_112556.2

*HNRNPD*: NM_031370.2; hnRNPD: NP_112738.1

*HNRNPF*: NM_001098208.1; hnRNPF: NP_001091678.1

*HNRNPH1*: NM_001257293.1; hnRNPH1: NP_001244222.1

*HNRNPH2*: NM_019597.4; hnRNPH2: NP_062543.1

*HNRNPH3*: NM_012207.2; hnRNPH3: NP_036339.1

*HNRNPK*: NM_031263.2; hnRNPK: NP_002131.2

*HNRNPR*: NM_001102398.1; hnRNPR: NP_001095868.1

*SYNCRIP*: NM_006372.4; SYNCRIP: NP_006363.4

*HNRNPU*: NM_031844.2; hnRNPU: NP_114032.2

*HNRNPUL1*: NM_007040.3; hnRNPUL1: NP_008971.2

*HNRNPUL2*: NM_001079559.2; hnRNPUL2: NP_001073027.1

PCBP1: NP_006187.2

PCBP2: NP_001122383.1

RMBX: NP_002130.2

RBMXL2: NP_055284.3

PTBP1: NP_114368.1

PTBP2: NM_021190.2; NP_067013.1

FUS: NP_004951.1

hnRNPL: NM_001533.2; NP_001524.2

hnRNPLL: NP_612403.2

hnRNPA1: NP_112420.1

hnRNPA1L: NP_001011724.1

hnRNPA3: NP_919233.1

hnRNPDL: NP_112740.1

hnRNPC: NP_002070910.1

hnRNPCL1: NP_001013643.1

hnRNPCL4: NP_00113953.1

RALY: NP_057951.1

RALYL: NP_001093862.1

hnRNPM: NP_005959.2
